# Strain-Resolved Dynamics of the Lung Microbiome in Patients with Cystic Fibrosis

**DOI:** 10.1128/mBio.02863-20

**Published:** 2021-03-09

**Authors:** Marija Dmitrijeva, Christian R. Kahlert, Rounak Feigelman, Rebekka L. Kleiner, Oliver Nolte, Werner C. Albrich, Florent Baty, Christian von Mering

**Affiliations:** a Department of Molecular Life Sciences, University of Zurich, Zurich, Switzerland; b Swiss Institute of Bioinformatics, Zurich, Switzerland; c Infectious Diseases and Hospital Epidemiology, Children’s Hospital of Eastern Switzerland, St. Gallen, Switzerland; d Infectious Diseases and Hospital Epidemiology, Cantonal Hospital St. Gallen, St. Gallen, Switzerland; e Pneumology and Sleep Medicine, Cantonal Hospital St. Gallen, St. Gallen, Switzerland; f Human Microbiology, Centre for Laboratory Medicine, St. Gallen, Switzerland; University of Toronto

**Keywords:** cystic fibrosis, longitudinal study, lung sputum, metagenomics, strain typing

## Abstract

In cystic fibrosis, dynamic and complex communities of microbial pathogens and commensals can colonize the lung. Cultured isolates from lung sputum reveal high inter- and intraindividual variability in pathogen strains, sequence variants, and phenotypes; disease progression likely depends on the precise combination of infecting lineages. Routine clinical protocols, however, provide a limited overview of the colonizer populations. Therefore, a more comprehensive and precise identification and characterization of infecting lineages could assist in making corresponding decisions on treatment. Here, we describe longitudinal tracking for four cystic fibrosis patients who exhibited extreme clinical phenotypes and, thus, were selected from a pilot cohort of 11 patients with repeated sampling for more than a year. Following metagenomics sequencing of lung sputum, we find that the taxonomic identity of individual colonizer lineages can be easily established. Crucially, even superficially clonal pathogens can be subdivided into multiple sublineages at the sequence level. By tracking individual allelic differences over time, an assembly-free clustering approach allows us to reconstruct multiple lineage-specific genomes with clear structural differences. Our study showcases a culture-independent shotgun metagenomics approach for longitudinal tracking of sublineage pathogen dynamics, opening up the possibility of using such methods to assist in monitoring disease progression through providing high-resolution routine characterization of the cystic fibrosis lung microbiome.

## Abstract (precis)

Cystic fibrosis patients frequently suffer from recurring respiratory infections caused by colonizing pathogenic and commensal bacteria. Although modern therapies can sometimes alleviate respiratory symptoms by ameliorating residual function of the protein responsible for the disorder, management of chronic respiratory infections remains an issue.

## INTRODUCTION

Cystic fibrosis (CF) is a monogenic, autosomal recessive, and life-shortening disease that predominantly affects the Caucasian population (1). The disease involves multiple organ systems but has its most severe consequences in the airways, where it leads to decreased mucociliary clearance followed by mucus plugging. Subsequently, the mucosal airways of CF patients are chronically inflamed and colonized by allochthonous microorganisms. The resulting respiratory symptoms include difficulty breathing, persistent cough, expectoration of sputum, and recurrent pulmonary infections. Respiratory failure accounts for more than half of CF patient deaths (2, 3). Nevertheless, improvements in CF management, such as antibiotic therapy and administration of mucolytic drugs, have increased the median life expectancy for patients, turning CF into a predominantly adult disorder (4). More recent therapies aim to directly ameliorate residual function of the protein encoded by the *CFTR* gene locus and have been shown to slow the rate of lung function decline in a subset of CF patients (5). Chronic respiratory infections, however, seem to persist even though respiratory symptoms improve (6). Therefore, improved characterization of persistent respiratory pathogens is needed to develop tailored therapies that control their composition and abundance.

Several common pathogens colonizing the lungs of CF patients are known. Pseudomonas aeruginosa is predominant in the adult CF population (2, 3). However, aggressive antimicrobial therapies aimed at reverting initial colonization by P. aeruginosa (7, 8) have recently led to a decline in its prevalence (2). Another key pathogen in CF is Staphylococcus aureus, which accounts for the majority of infections in young patients and has become increasingly more prevalent among all CF patients (2, 3). Other pathogens recognized in CF include members of the Burkholderia cepacia complex, mycobacteria such as Mycobacterium avium and Mycobacterium abscessus, Stenotrophomonas maltophilia, and members of the *Achromobacter* genus (2, 3, 9). Although the latter pathogens are present in a small fraction of CF patients, they are often multidrug resistant and, thus, challenging with regard to the treatment options in the clinic. Finally, anaerobic bacteria such as members of the *Prevotella* genus have also been identified in CF patient sputum using specialized culture techniques (10), but these typically are not assessed during routine clinical diagnosis, and their role as pathogens in CF patients has yet to be defined.

Culture-independent approaches are increasingly complementing and expanding on the findings of traditional microbiology approaches. For instance, studies using sequencing to characterize the lung microbiome have noted the presence of anaerobic bacteria not recognized as typical CF pathogens, such as *Prevotella* and *Veillonella*, in a sizable portion of the patients (11–13). In addition, culture-independent approaches uncovered a high level of variability across the lung microbiomes of CF patients (13–18). In late-stage patients, however, the microbiome generally tends to be lower in diversity and becomes dominated by one or a few of the commonly recognized CF pathogens (13, 14, 17). Several efforts have compared patient-matched samples from different clinical states but have not found significant reproducible changes between samples taken at baseline and at exacerbation, which suggests that the CF lung microbiome is resilient over time (11, 12, 14–16). Most culture-independent studies of the lung microbiome in CF, however, have been performed using 16S rRNA sequencing and, thus, provide only limited insights into the functions or strain identities of lung microbial communities.

Whole-genome sequencing (WGS) and metagenome sequencing improve on the limited taxonomic and functional resolution of 16S rRNA sequencing. Metagenomics allows us to survey bacterial, viral, and fungal populations at once, giving a more complete picture of microbial relative abundances in the CF lung microbiome (17, 19). Consequently, a larger portion of microbiome inhabitants can be classified at the species level (17), and prominent CF pathogens have been classified at the strain level (17, 18, 20). Moreover, multiple subpopulations of specific pathogens have been detected in CF through metagenome sequencing (17, 18). However, so far only single reference points per patient were typically sequenced, limiting haplotype deconvolution and preventing insights into the temporal dynamics of these lineages.

Here, we describe longitudinal sputum sampling in CF patients over the course of one and a half years, conducting metagenomics sequencing of spontaneously expectorated sputum at multiple time points. The aim of the study was to investigate the advantages of collecting longitudinal data of CF patients for monitoring and characterizing lineage successions *in situ*. We successfully classified most of the lung microbiome members at the species and genus level and confirmed the presence of pathogens identified during routine clinical diagnosis. Importantly, we show how longitudinal metagenomics data can be used to deconvolute distinct lineage variants of the same species within a given patient. We introduce an assembly-free approach that can delineate nearly complete, lineage-specific genomes even when their sequence divergence is fairly low. Our study introduces culture-independent methods that can be used in the future for monitoring pathogen lineages in CF.

## RESULTS

### Patient-specific lung microbiomes.

**FIG 1:**
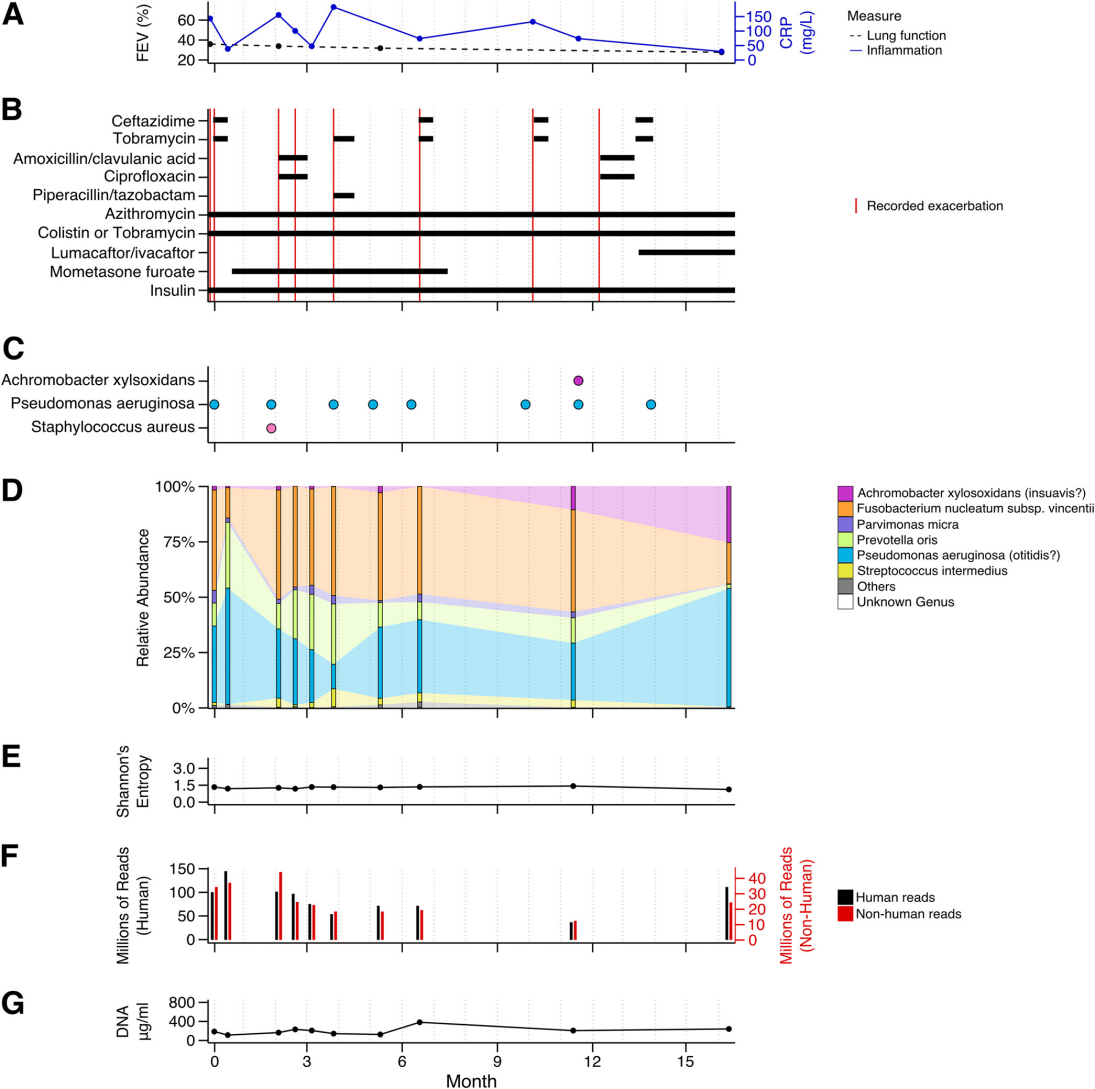
Study report of patient CFR11 displaying the dynamics of multiple parameters over time. (A) Percent forced expiratory volume (FEV) (black) and concentration of C-reactive protein (CRP) (blue), with actual measurements shown as dots. (B) Medication assigned to the patient during the course of the study and recorded exacerbation events (in red). (C) Bacteria identified in the clinical microbiology laboratory. (D) Relative abundance profiles generated by mOTUs, with actual measurements shown as bars. Selected species and their corresponding genera with more than 5% relative abundance at at least one time point are shown color-coded. Less abundant species are grouped into “Others” (gray). Taxa that could not be identified by mOTUs on the genus level are grouped into “Unknown Genus” (white). (E) Shannon’s diversity index (entropy) calculated based on the relative abundance profiles generated by mOTUs, with actual measurements shown as dots. (F) Number of reads per sample. Human reads are indicated in black and plotted on the left axis. Reads that did not concordantly map to the human genome are depicted in red and plotted on the right axis. (G) Concentration of total DNA isolated from patient sputum, with actual measurements shown as dots.

We monitored four CF patients selected from a larger cohort over the course of 19 months (Fig. 1; see also Fig. S1, S2, and S3 in the supplemental material). A summary of patient information, clinical parameters, and prescribed medication is available in Data Set S1 at https://string-db.org/suppl/Dataset_S1_Strain-resolved_Microbiome_Dynamics_in_Cystic_Fibrosis.xlsx. During the course of our study, the patients produced sputum spontaneously, either during routine clinical check-ups or during exacerbations. In total, 25 samples were collected. We extracted total DNA from the collected sputum, enriched for nonmethylated DNA, and sequenced it using the Illumina HiSeq 4000 platform. Sequencing depth varied between 21 million reads and 179 million reads (Fig. 1F and Fig. S1F, S2F, and S3F). Human DNA constituted between 70% and 93% of all reads. We observed no significant associations between the total DNA concentration in the sample and the sequencing depth or the fraction of nonhuman reads. Nonhuman DNA predominantly originated from bacteria; viruses (including bacteriophages) accounted for, at most, 1.5% of reads, and fungi accounted for, at most, 0.15% of the reads (as profiled by MiCoP) (21). More than 95% percent of the bacteria at each time point could be identified to at least the genus level using the mOTUs software (22) (Fig. 1D and Fig. S1D, S2D, S3D, and Data Set S1 at the URL mentioned above, mOTUs), indicating that the lung microbiomes of these patients largely consisted of previously characterized bacterial clades.

10.1128/mBio.02863-20.1 FIG S1 Study report of patient CFR06 displaying the dynamics of multiple parameters over time. (A) Percent forced expiratory volume (FEV) (black) and concentration of C-reactive protein (CRP) (blue), with actual measurements shown as dots. (B) Medication assigned to the patient during the course of the study and recorded exacerbation events (in red). (C) Bacteria identified in the clinical microbiology laboratory. (D) Relative abundance profiles generated by mOTUs, with actual measurements shown as bars. Selected species and their corresponding genera with more than 5% relative abundance at at least one time-point are shown color-coded. Less abundant species are grouped into “Others” (gray). Taxa that could not be identified by mOTUs on the genus level are grouped into “Unknown Genus” (white). (E) Shannon’s diversity index (entropy) calculated based on the relative abundance profiles generated by mOTUs, with actual measurements shown as dots. (F) Number of reads per sample. Human reads are indicated in black and plotted on the left axis. Reads that did not concordantly map to the human genome are depicted in red and plotted on the right axis. (G) Concentration of total DNA isolated from patient sputum, with actual measurements shown as dots. Download FIG S1, PDF file, 0.8 MB. Copyright © 2021 Dmitrijeva et al. 2021 Dmitrijeva et al. https://creativecommons.org/licenses/by/4.0/ This content is distributed under the terms of the Creative Commons Attribution 4.0 International license.

10.1128/mBio.02863-20.2 FIG S2 Study report of patient CFR07 displaying the dynamics of multiple parameters over time. (A) Percent forced expiratory volume (FEV) (black) and concentration of C-reactive protein (CRP) (blue), with actual measurements shown as dots. (B) Medication assigned to the patient during the course of the study and recorded exacerbation events (in red). (C) Bacteria identified in the clinical microbiology laboratory. (D) Relative abundance profiles generated by mOTUs, with actual measurements shown as bars. Selected species and their corresponding genera with more than 5% relative abundance at at least one time-point are shown color-coded. Less abundant species are grouped into “Others” (gray). Taxa that could not be identified by mOTUs on the genus level are grouped into “Unknown Genus” (white). (E) Shannon’s diversity index (entropy) calculated based on the relative abundance profiles generated by mOTUs, with actual measurements shown as dots. (F) Number of reads per sample. Human reads are indicated in black and plotted on the left axis. Reads that did not concordantly map to the human genome are depicted in red on the right axis. (G) Concentration of total DNA isolated from patient sputum, with actual measurements shown as dots. Download FIG S2, PDF file, 0.3 MB. Copyright © 2021 Dmitrijeva et al. 2021 Dmitrijeva et al. https://creativecommons.org/licenses/by/4.0/ This content is distributed under the terms of the Creative Commons Attribution 4.0 International license.

10.1128/mBio.02863-20.3 FIG S3 Study report of patient CFR09 displaying the dynamics of multiple parameters over time. (A) Percent forced expiratory volume (FEV) (black) and concentration of C-reactive protein (CRP) (blue), with actual measurements shown as dots. (B) Medication assigned to the patient during the course of the study and recorded exacerbation events (in red). (C) Bacteria identified in the clinical microbiology laboratory. (D) Relative abundance profiles generated by mOTUs, with actual measurements shown as bars. Selected species and their corresponding genera with more than 5% relative abundance at at least one time-point are shown color-coded. Less abundant species are grouped into “Others” (gray). Taxa that could not be identified by mOTUs on the genus level are grouped into “Unknown Genus” (white). (E) Shannon’s diversity index (entropy) calculated based on the relative abundance profiles generated by mOTUs, with actual measurements shown as dots. (F) Number of reads per sample. Human reads are indicated in black and plotted on the left axis. Reads that did not concordantly map to the human genome are depicted in red and plotted on the right axis. (G) Concentration of total DNA isolated from patient sputum, with actual measurements shown as dots. Download FIG S3, PDF file, 0.4 MB. Copyright © 2021 Dmitrijeva et al. 2021 Dmitrijeva et al. https://creativecommons.org/licenses/by/4.0/ This content is distributed under the terms of the Creative Commons Attribution 4.0 International license.

Lung microbiome compositions showed marked differences between the four patients (Fig. S4). For instance, the lung microbiome profile of patient CFR06 contained between 60% and 93% of anaerobic bacteria, including the oral anaerobes *Prevotella*, *Parvimonas*, and *Fusobacterium*, and was the only patient devoid of any detectable *Pseudomonas* (Fig. S1D). Typical CF pathogens identified by the clinical microbiology laboratory, A. xylosoxidans, H. influenzae, and S. aureus (Fig. S1C), and their corresponding genera accounted for less than a fourth of the bacterial content (Fig. S1D). This patient was several years younger than the other subjects and displayed a milder form of CF, with the highest average forced expiratory volume (FEV), a measure of lung function (95% confidence interval [CI], 56.8% ± 6.4% versus 30.7% ± 0.7% in CFR07, 40.0% ± 3.1% in CFR09, 32.5% ± 3.3% in CFR11) (Fig. S1A). Around 7 months into the study, the patient had an exacerbation that was treated with a combination of piperacillin-tazobactam and intravenous tobramycin (Fig. S1B). Following this event, the lung microbiome composition of CFR06 experienced a major shift: Prevotella buccae, S. aureus, and A. xylosoxidans*/insuavis* all decreased in relative abundance. Concomitantly, Prevotella oris, Fusobacterium nucleatum, and Gemella morbillorum increased in relative abundance (Fig. S1D).

10.1128/mBio.02863-20.4 FIG S4 Principal-component analysis of Manhattan distances between patient samples. The plot on the left shows the first three components (94% of the variation). The plot on the right shows the first two components (87% of the variation) with time points at which the samples were collected. Colors: CFR06 (blue), CFR07 (green), CFR09 (purple), and CFR11 (orange). Download FIG S4, PDF file, 0.3 MB. Copyright © 2021 Dmitrijeva et al. 2021 Dmitrijeva et al. https://creativecommons.org/licenses/by/4.0/ This content is distributed under the terms of the Creative Commons Attribution 4.0 International license.

In contrast, the lungs of patients CFR07 and CFR09 were colonized predominantly by P. aeruginosa (Fig. S2C and D and S3C and D), with samples from these patients clustering together (Fig. S4). Patient CFR07 displayed a stable clinical phenotype, with no exacerbations recorded during the course of the study, and retained FEV at 30% (Fig. S2A and B). P. aeruginosa accounted for more than 90% of all bacteria in this patient’s lung microbiome, resulting in the lowest microbiome diversity of all examined patients (average Shannon’s diversity index with 95% CI, 0.48 ± 0.09 versus 2.29 ± 0.44 in CFR06, 1.79 ± 0.93 in CFR09, and 1.29 ± 0.06 in CFR11) (Fig. S2E). The lung microbiome of patient CFR09 contained, in addition to P. aeruginosa, up to 17.5% and 9.2% of the genera *Prevotella* and *Veillonella*, respectively, and various low-abundance genera that comprised up to 35% of the lung microbiome (Fig. S3D).

Finally, patient CFR11 presented the most severe course of disease (Fig. 1) and died shortly after study completion. The patient experienced multiple exacerbations, accompanied by high levels of inflammation, with FEV gradually declining from 36% to 28% (Fig. 1A and B). The lung microbiome of CFR11 was dominated by P. aeruginosa (Fig. 1C and D) and the oral anaerobes *P. oris* and F. nucleatum (Fig. 1D). In addition, Parvimonas micra, Streptococcus intermedius, and A. xylosoxidans*/insuavis* were present in lower relative abundances, with the fraction of *Achromobacter* increasing at later time points (Fig. 1D), to the point of also being detected using standard clinical microbiology procedures (Fig. 1C). Thus, in three of the patients, the most abundant bacteria remained the same throughout the course of the study, and only CFR06 displayed a major sustained shift in lung microbiome composition (Fig. S4). From all identified bacteria, the most relevant from a clinical perspective were A. xylosoxidans (identified in two patients, CFR06 and CFR11) and P. aeruginosa (identified in three patients, CFR07, CFR09, and CFR11). Therefore, we set out to assess to what extent cultivation-independent sequencing would allow us to classify these pathogens in more detail.

### Strain-level classification of *Achromobacter*.

Seven of the sputum samples contained sufficient reads to provide a 2-fold median coverage of the A. xylosoxidans pangenome (one sample from CFR06 and six samples from CFR11), and the corresponding assembled contigs showed more than 80% expected genome completeness according to CheckM (23) (Data Set S1 at https://string-db.org/suppl/Dataset_S1_Strain-resolved_Microbiome_Dynamics_in_Cystic_Fibrosis.xlsx, assembly reports). From a selection of 22 fully sequenced A. xylosoxidans reference genomes, A. xylosoxidans FDAARGOS_147, a strain isolated from a patient at Children’s National Hospital in Washington, DC, was the only genome with more than 50% gene family overlap with the *Achromobacter* contigs from the patients (Fig. S5A and B). Therefore, we decided to compare marker gene sequence identity to place our samples within the *Achromobacter* genus.

10.1128/mBio.02863-20.5 FIG S5 Strain identification for clinically relevant pathogens A. xylosoxidans and P. aeruginosa. (A) t-SNE plot based on A. xylosoxidans gene family presence/absence profiles generated by PanPhlAn. (B) Heatmap showing the percentage of gene family overlap calculated based on the gene family presence/absence profiles from panel A. (C) t-SNE plot based on P. aeruginosa gene family presence/absence profiles generated by PanPhlAn. (D) Midpoint-rooted phylogenetic tree of selected P. aeruginosa strains based on 10 gene marker sequences from mOTUs. Colors: CFR06 (blue), CFR07 (green), CFR09 (purple), CFR11 (orange), and reference genomes (gray). Download FIG S5, PDF file, 1.0 MB. Copyright © 2021 Dmitrijeva et al. 2021 Dmitrijeva et al. https://creativecommons.org/licenses/by/4.0/ This content is distributed under the terms of the Creative Commons Attribution 4.0 International license.

**FIG 2:**
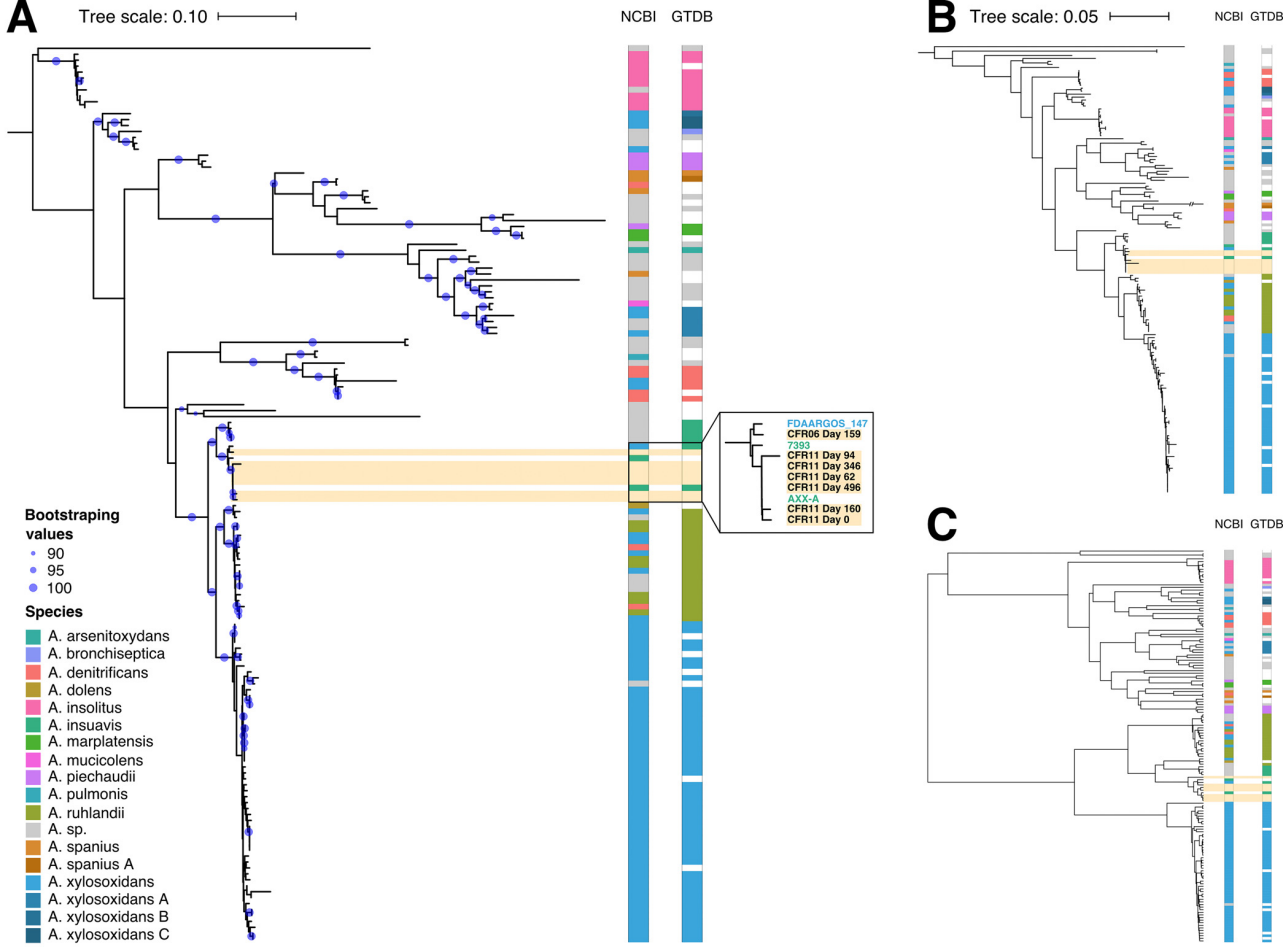
Strain-typing of patient-specific *Achromobacter* in the context of a sequence-based phylogeny of the genus. (A) Maximum-likelihood tree based on sequences of 10 single-copy genes used by mOTUs. Colors on the right of the tree depict species assignments according to NCBI and GTDB taxonomies and apply to panels B and C as well. Blue circles indicate branch confidence values (≥90) based on 100 bootstraps of the tree. (B) Maximum-likelihood tree based on sequences of seven housekeeping genes from the *Achromobacter* MLST database. (C) UPGMA clustering of pairwise average nucleotide identities of the comprising *Achromobacter* genomes.

From 144 *Achromobacter* genomes in the NCBI genome database (November 2018) (24), *Achromobacter* genus trees were constructed using the sequences from the 10 single-copy genes used by mOTUs, sequences from the seven genes used in standard *Achromobacter* MLST (25), or pairwise genome average nucleotide identities (see Materials and Methods). The three genus trees were more consistent with each other (average normalized Robinson-Foulds distance, 0.62) than to a phylogenetic tree informed by a single marker gene, such as 16S rRNA (average normalized Robinson-Foulds distance, 0.90). All three trees revealed some discrepancies with the NCBI taxonomy (Fig. 2, NCBI columns). Out of the eight clades containing more than one genome, only *A. marplatensis* and *A. insolitus* were monophyletic in all three trees. Conversely, our analyses clustered together genomes assigned to different species with more than 90% bootstrap support. For example, one well-supported cluster contained genomes from *A. ruhlandii*, *A. denitrificans*, and A. xylosoxidans (Fig. 2A). As has been noted previously, taxonomic classification within the *Achromobacter* genus has inconsistencies (26). The Genome Taxonomy Database (GTDB) is a recent effort to redefine prokaryotic taxonomy and improve on such inconsistencies in species assignment (27). To determine whether this approach could be of help here, we downloaded the species assignments from GTDB (as of April 2019). Indeed, seven of the nine multigenome *Achromobacter* clades defined by GTDB were monophyletic in all three trees, and all nine clades were monophyletic in the mOTU tree (Fig. 2, GTDB columns).

On all three *Achromobacter* genus trees, we observed that our patient-derived *Achromobacter* genomes and A. xylosoxidans FDAARGOS_147 clustered with two *A. insuavis* genomes. The lineage in patient CFR11 was 99.99% identical to *A. insuavis* AXX-A, a strain observed at the Laboratory of Bacteriology at the Faculty of Medicine in Dijon, France (Fig. 2A, zoom-in). The lineage in patient CFR06 clustered with A. xylosoxidans FDAARGOS_147 and was 99.24% identical to it (Fig. 2A, zoom-in). Taken together, these results indicated that the clinical laboratory misidentified this pathogen, incorrectly reporting A. xylosoxidans instead of *A. insuavis*.

### Strain-level classification of P. aeruginosa.

Next, we asked how well strain identification performs in the case of P. aeruginosa, a species for which more comprehensive reference information is available. Patients CFR07, CFR09, and CFR11 all carried *Pseudomonas* in sufficient amounts to allow 99% estimated genome coverage (Data Set S1 at https://string-db.org/suppl/Dataset_S1_Strain-resolved_Microbiome_Dynamics_in_Cystic_Fibrosis.xlsx, assembly reports). To identify the lineages, we compared our samples to a representative set of 359 P. aeruginosa genomes based on gene family presence/absence profiles (28) (Fig. S5C) and marker gene sequence identity (Fig. S5D). Patients CFR09 and CFR11 harbored a lineage with single-copy gene sequences 100% identical to those of P. aeruginosa PAER4_119 (first sampled in Poland), although some differences in gene family content were present (Fig. S5C and D). The samples from CFR07, however, contained gene families distinct from those of the other patients and clustered with other reference genomes (Fig. S5C). Indeed, the tree based on marker genes revealed that CFR07 is infected by a new lineage that was, at most, 99.73% identical to the genomes in the representative set (Fig. S5D).

Our work with *Pseudomonas* indicated that more than one variant of the lineage was likely present in some of the patient samples, most notably in CFR11. Both PanPhlAn and CheckM presented corresponding warnings but could not further delineate these lineage variants.

### Delineation of P. aeruginosa lineage variants without cultivation or genome assembly.

**FIG 3:**
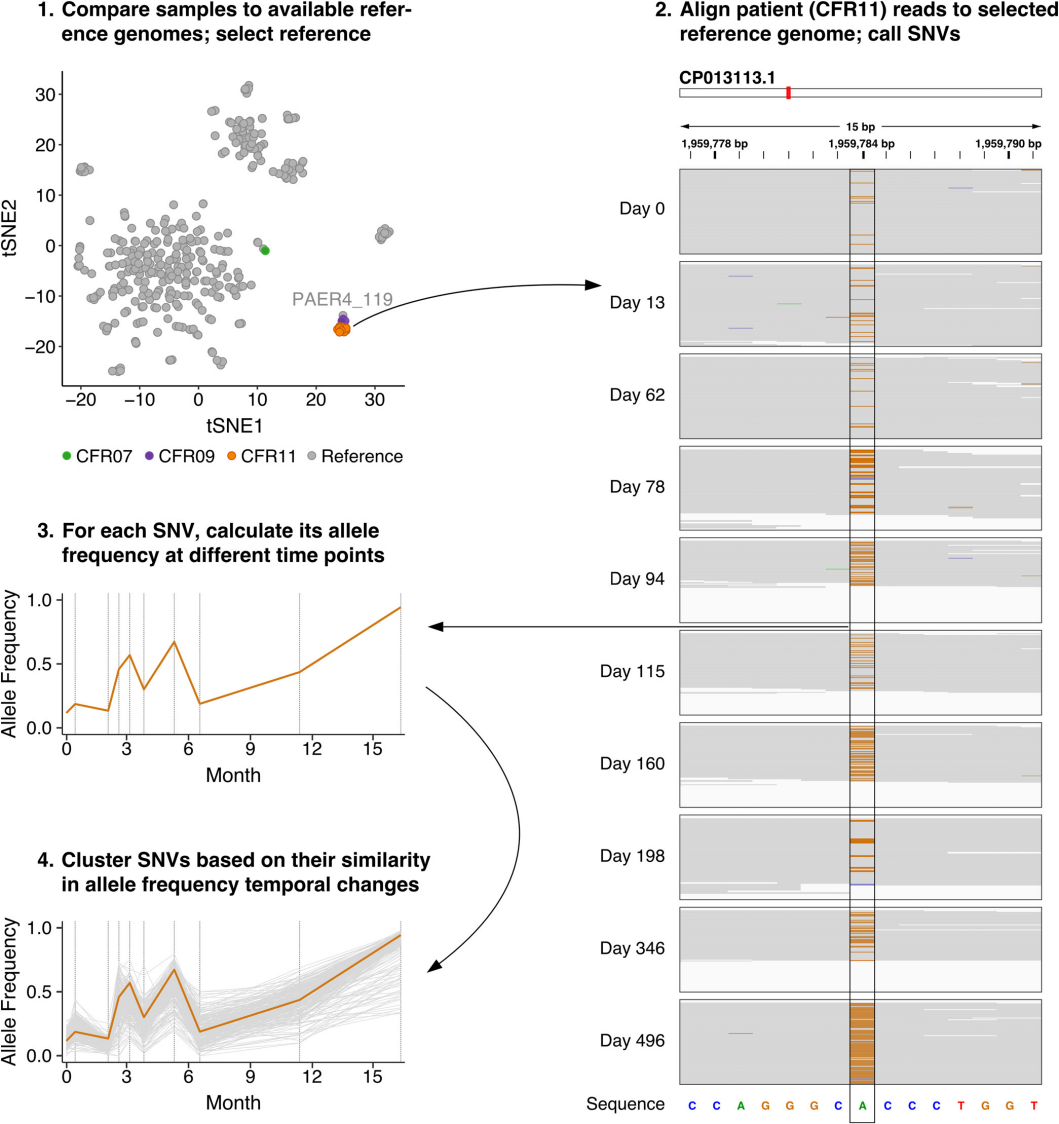
Identification of lineage variants through assessment of temporal changes in SNV allele frequencies in the metagenomics data of patient CFR11. (Step 1) Selection of a reference genome based on generated gene family presence/absence profiles. (Step 2) Read mapping of CFR11 samples to the reference genome (CP013113.1). A pile-up of a selected region containing an SNV (1,959,777 to 1,959,791 bp) is shown for every time point. The reference sequence is displayed on the bottom. Gray indicates read base pairs that are identical to the reference sequence. Orange indicates that a substitution to guanine has occurred. (Step 3) The change in allele frequency over time for the selected SNV. (Step 4) A group of SNVs that show a similar pattern of temporal changes in allele frequencies. The selected SNV is depicted in orange. The explicit steps performed and tools used in this approach can be found in a flow chart in Fig. S6.

To distinguish and track P. aeruginosa lineage variants in patient CFR11, we took advantage of the repeated samplings in our time series data. Under the assumption that the relative abundances of competing P. aeruginosa populations in a patient would vary over time, any population-specific sequence variants should similarly vary over time. This would allow reconstructing constituent genomes through clustering sequence variants by their shared temporal behavior. To test this approach, we mapped all apparent *Pseudomonas* reads from patient CFR11 to the closest reference genome (P. aeruginosa PAER4_119), which served as a scaffold (Fig. 3, steps 1 and 2). We then called single-nucleotide variants (SNVs) using metaSNV (29) and determined their allele frequencies at each time point (Fig. 3, steps 2 and 3). Finally, we determined clusters of SNVs displaying similar changes in allele frequencies over time (Fig. 3, step 4).

10.1128/mBio.02863-20.6 FIG S6 Flow chart depiction of steps performed to identify lineage variants in the study. The names of databases used in the analysis are depicted in orange. The names of tools used in the analysis are depicted in blue. Download FIG S6, PDF file, 0.4 MB. Copyright © 2021 Dmitrijeva et al. 2021 Dmitrijeva et al. https://creativecommons.org/licenses/by/4.0/ This content is distributed under the terms of the Creative Commons Attribution 4.0 International license.

**FIG 4:**
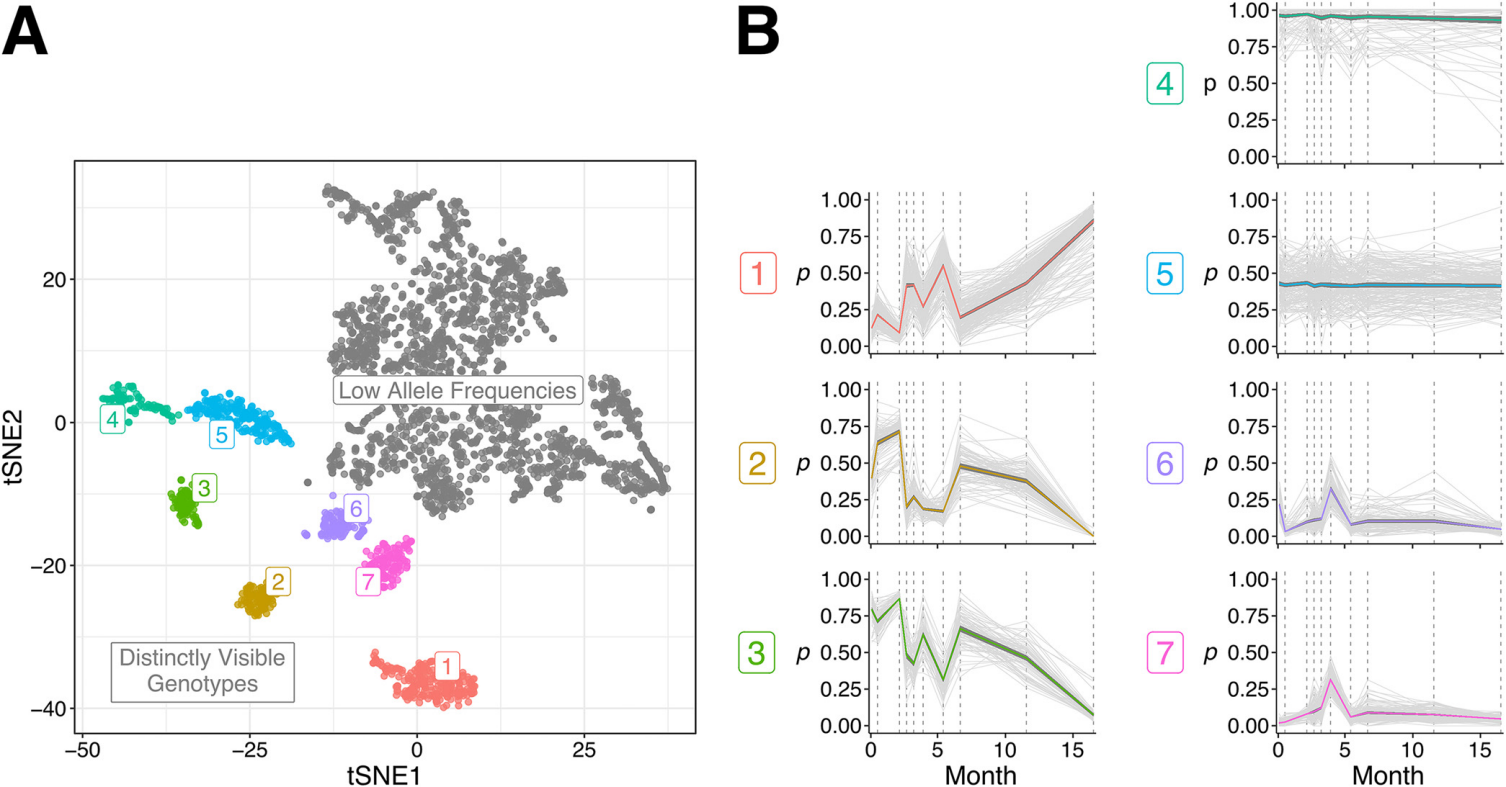
Clustering of SNVs detected in patient CFR11 based on their temporal changes in allele frequencies. (A) A t-SNE plot depicting the clustering pattern of 3,079 SNVs called in patient CFR11. Most SNVs occur at low allele frequencies (gray). The remaining SNVs form seven distinctly visible genotypes that are labeled and colored accordingly. (B) Changes in the allele frequencies (*p*) of SNVs belonging to each distinct genotype over time. The colored line indicates mean allele frequency of the genotype. Dark gray ribbons indicate the 95% confidence intervals.

A total of 3,451 SNVs were called, of which 3,079 had allele frequencies detected at every time point, with at least one allele frequency not equal to one. Repeated t-distributed stochastic neighbor embedding (t-SNE) runs at slightly varying settings consistently yielded seven distinct clusters of SNVs in addition to a pool of lower-frequency SNVs that could not be reliably clustered (Fig. 4A). Of the seven clusters, each showed a clearly distinct pattern of allele frequencies over time (Fig. 4B). Cluster 3 appeared to be a linear sum of clusters 2 and 6 (*P* < 2.2E−16; comparison between the sum of SNV allele frequencies from the aforementioned clusters over 10 time points to that of the same SNVs but with the time points shuffled) and to inversely correlate with cluster 1 (*P* < 2.2E−16), not differing from it significantly in the extent of temporal variation (*P* = 0.38; comparison of standard deviations in individual SNV allele frequencies between clusters). Clusters 6 and 7 followed a somewhat shared pattern over time, with the exception of the first time point (*P* < 2.2E−16), and did not significantly differ in their extent of temporal variation (*P* = 0.10). Cluster 5 exhibited significantly less temporal variation than cluster 6 (*P* = 3.4E−25), and cluster 4 exhibited the least temporal variation.

To investigate the clusters more carefully, we plotted the spatial positioning of the cluster-specific SNVs in the reference genome (Fig. S7A). We found no association between the distance of SNVs from the same cluster on the chromosome and the similarity in their temporal profiles (Fig. S7C), indicating that the differences in allele frequency patterns were not simply due to recombination of selected genomic regions containing multiple SNVs. Neighboring SNVs from clusters 1, 2, 3, 6, and 7 were located within the expected range of distances, indicating homogeneous distribution, but neighboring SNVs from clusters 4 and 5 were closer to each other than expected (*P* < 1E−04), indicating the concentration of SNVs in selected genomic regions (Fig. S7B). Together with the fact that these clusters exhibited less SNV temporal profile cohesiveness than clusters 1, 2, 3, 6, and 7 (data not shown), we interpret such SNVs to reflect intragenomic polymorphisms in relation to the reference genome (e.g., at tandem-repeat regions) that were artifactually clustered together. Hence, clusters 4 and 5 were discarded. Considering the remaining clusters, the most parsimonious interpretation of the data appears to be the presence of three distinct P. aeruginosa lineage variants in the patient (reflected in clusters 1, 2, and 6/7, respectively). In this scenario, cluster 3 would consist of SNVs that are ancestrally shared between two of the variants and whose frequencies reflect the sum of their relative abundances.

10.1128/mBio.02863-20.7 FIG S7 Assessment of positional bias in the distribution of SNVs from the Fig. 4 SNV clusters from patient CFR11 in the P. aeruginosa genome. (A) Plot depicting the distribution of SNVs along the P. aeruginosa PAER4_119 (CP013113.1) chromosome. Each dot represents an SNV assigned to one of the seven clusters and is colored according to its cluster in Fig. 4. (B) Quantification of positional bias in the distribution of SNVs on the chromosome. Vertical lines indicate the actual median distance between neighboring SNVs. Background distributions of median distances between neighboring SNVs (in gray) are generated by randomly selecting SNV groups of the same size as the considered cluster from the seven-cluster SNV pool. The procedure is repeated 10,000 times for each cluster. *P* values reflect the probability of the actual distance lying within the background distribution. (C) Temporal profile similarity of SNV pairs versus the distance between these SNVs (for selected clusters 1, 2, and 7). Results are represented as a two-dimensional histogram in which yellow indicates a large number of SNV pairs and blue indicates a small number of SNV pairs. No significant correlations between temporal profile similarity and chromosomal distance are found. Download FIG S7, PDF file, 1.0 MB. Copyright © 2021 Dmitrijeva et al. 2021 Dmitrijeva et al. https://creativecommons.org/licenses/by/4.0/ This content is distributed under the terms of the Creative Commons Attribution 4.0 International license.

Because t-SNE is a nondeterministic algorithm, we sought to validate our observations. Therefore, we used the called SNVs to perform principal-component analysis (PCA) combined with hierarchical clustering and to run DESMAN, a tool developed for grouping SNVs into haplotypes by assessing the variation of nucleotide base frequencies across samples and by using a Bayesian model to resolve possible sequencing errors and SNVs that are shared between more than one strain (30). The clusters generated based on PCA were largely consistent, the only deviation being a merging of t-SNE clusters 6 and 7 (Fig. S8A and B). The three haplotypes yielded by DESMAN coincided with clusters 1, 2, and 6 (Fig. S8C and D). Thus, we could validate the majority of SNVs that were clustered together in t-SNE (Fig. S8E). In addition, we could confirm via additional long-read sequencing that SNVs that were observed to cluster together by all three methods indeed occurred on the same DNA molecule significantly more often than expected based on their individual allele frequencies alone (*P* < 1E−04) (Fig. S9). Taken together, our results suggest the coexistence of three lineage variants that, notably, would have been impossible to distinguish using the 16S rRNA gene alone (Fig. S10). Likewise, at the observed pairwise divergence of less than 0.01% between the variant genomes, traditional genome assembly approaches also would likely not be able to distinguish these (31).

10.1128/mBio.02863-20.8 FIG S8 Validation of detected P. aeruginosa SNV clusters in patient CFR11. (A) Plot of the first two principal components (95% of the variation) generated based on the complete allele frequency table. SNVs are clustered into eight clusters based on the first three components (97% of variation), and dots are colored according to cluster assignment. (B) Projections of the clusters shown in panel A on the t-SNE plot from Fig. 4. (C) Temporal relative abundance profiles of the three lineage variants detected by DESMAN. Dots depict individual runs (*n* = 10), and the line shows the average values. (D) Projection of SNVs assigned to each of the three lineage variants by DESMAN on the t-SNE plot from Fig. 4. Colors indicate lineage variant assignment: red (variant 1), yellow (variant 2), and purple (variant 3). (E) Venn diagrams depicting the overlap of SNVs assigned to each of the three variants by t-SNE clustering, principal component clustering, and DESMAN. Download FIG S8, PDF file, 1.2 MB. Copyright © 2021 Dmitrijeva et al. 2021 Dmitrijeva et al. https://creativecommons.org/licenses/by/4.0/ This content is distributed under the terms of the Creative Commons Attribution 4.0 International license.

10.1128/mBio.02863-20.9 FIG S9 Validation of detected P. aeruginosa lineage variants in patient CFR11 via long-read sequencing. (A) An example of a read spanning four SNVs that are diagnostic for specific lineage variants. A haplotype is assigned based on the combination of base pairs at the indicated positions in the read. This haplotype is either compatible with one of the proposed lineage variants or labeled “incompatible.” The number of observed long reads corresponding to each defined haplotype can be seen in panel B for the day 94 sample and panel C for the day 346 sample. Vertical lines indicate the observed number of reads. Background distributions of read numbers (in gray) are created by generating random haplotypes based on metaSNV allele frequencies from the corresponding day. The procedure is repeated 10,000 times for all reads spanning at least two diagnostic SNVs. *P* values reflect the probability of the observed number of reads lying within the background distribution. Download FIG S9, PDF file, 0.5 MB. Copyright © 2021 Dmitrijeva et al. 2021 Dmitrijeva et al. https://creativecommons.org/licenses/by/4.0/ This content is distributed under the terms of the Creative Commons Attribution 4.0 International license.

10.1128/mBio.02863-20.10 FIG S10 SNV detection in the 16S rRNA sequence of P. aeruginosa. (A) A snapshot from Integrative Genome Viewer showing 16S rRNA coverage profiles from all time points of patient CFR11. Reference sequence shown on the bottom: adenine (green), cytosine (blue), guanine (orange), and thymine (red). Deviations from the reference sequence shown as colorful stripes on the coverage profiles. (B) Temporal dynamics of the fraction of reads containing detected SNVs. SNVs are depicted in different colors, the number corresponding to location in the 16S sequence. The relative abundances of the three lineage variants detected by DESMAN are shown as dotted lines. SNVs at positions 769, 773, 1004, and 1093 are located in regions with higher coverage and closely resemble the gain in coverage. Download FIG S10, PDF file, 0.5 MB. Copyright © 2021 Dmitrijeva et al. 2021 Dmitrijeva et al. https://creativecommons.org/licenses/by/4.0/ This content is distributed under the terms of the Creative Commons Attribution 4.0 International license.

Subsequently, we focused on SNVs that were assigned to the same cluster or haplotype by all three methods (Fig. S8E). Out of the 563 SNVs from all three lineage variants, 502 overlapped a gene in P. aeruginosa PAER4_119, with 459 genes containing at least one SNV (Data Set S1 at https://string-db.org/suppl/Dataset_S1_Strain-resolved_Microbiome_Dynamics_in_Cystic_Fibrosis.xlsx, diagnostic SNV genes). We next wondered whether genes known to be mutated in CF (32) would be preferentially mutated in our lineage variants. Only variant 2 had a borderline significant enrichment of mutations in these genes compared to the rest of the genome (*P* = 0.03, Fisher’s exact test), while variants 1 and 3 had no enrichment compared to the rest of the genome (*P* values of 0.21 and 0.15, respectively, Fisher’s exact test).

At least 100 SNVs separated each lineage variant from the other (Table 1). The rate of mutations in P. aeruginosa has been estimated to be, at most, 5.5 SNVs per year (33–35), unless a hypermutator phenotype develops (34, 36). To test for potential hypermutator mutations, we mapped reads to the six DNA repair genes known to be affected in hypermutator strains: *mutS*, *mutL*, *uvrD*, *mutM*, *mutY*, and *mutT* (35–38). We detected only one mutation that might disrupt gene function, a frameshift deletion in the *mutS* gene. However, this mutation was observed only in reads corresponding to lineage variant 1 (not variant 2 or 3), and the mutation was positioned toward the 3′ end of the gene, close to the stop codon of the predicted open reading frame.

**TABLE 1:** Number of SNVs consistently clustered by three different approaches (t-SNE, PCA, and DESMAN)

SNV type	No. of SNVs
Lineage variant 1 specific	204
Lineage variant 2 specific	106
Lineage variant 3 specific	186
Shared between 2 and 3	67

### Detection of P. aeruginosa variant-specific structural genome differences.

To determine whether longitudinal metagenomics sequencing provides sufficient evidence to detect large-scale genomic variation between lineage variants, we mapped reads to the reference P. aeruginosa PAER4_119 genome and calculated the average read coverage for windows of 1,000 bp along the genome. After normalizing for higher read coverage around the origin of replication (39), any large-scale genomic differences between lineage variants should become evident as time point-dependent deviations in read coverage.

**FIG 5:**
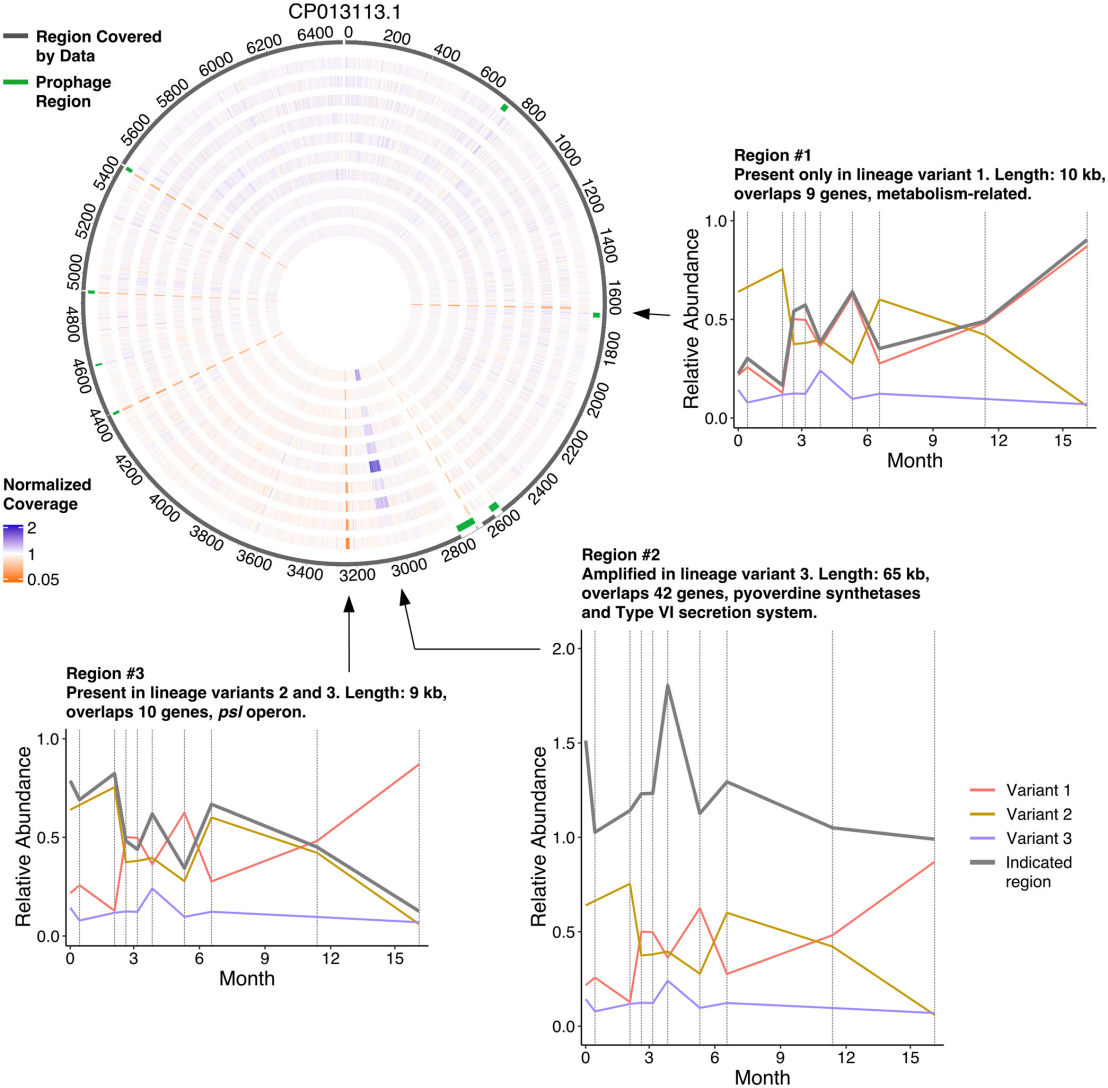
Assessment of temporal variation in the coverage of specific regions in the genome of P. aeruginosa in patient CFR11. The circos diagram provides an overview of the genome coverage profiles with chromosomal coordinates in kb. The dark gray outer circle depicts regions of the reference genome that have a coverage of at least 5% from the average coverage at at least one time point. The second outermost circle depicts detected phage regions in green. The remaining circles depict normalized coverage profiles for each of the 10 time points sampled for patient CFR11 (innermost, day 0; outermost, day 496). Orange regions indicate lower than average coverage, and purple-blue regions indicate higher than average coverage. Insets highlight three regions that display variant-specific coverage profiles. Each variant is depicted in a distinct color, and the average coverage of the selected region is depicted in gray.

Indeed, the mean coverage in 70 windows was at least two standard deviations from the overall mean coverage, combining into seven regions of the genome that spanned more than two windows each (Fig. 5, circos diagram). Of these, the regions located around 4.4 Mbp, 4.9 Mbp, and 5.5 Mbp coincided with predicted phage sequences; structural variations at phage insertions are to be expected. A fourth region around 2.7 Mbp showed inconsistent and overall low coverage in our sample data and was not considered further. This left three regions of interest. Manual inspection allowed us to pinpoint region borders more precisely at 1,639,504 bp to 1,649,747 bp and 3,024,303 bp to 3,088,958 bp for two of the regions. The third region was revealed to be composite, its borders at 3,228,483 bp to 3,235,055 bp and 3,241,118 bp to 3,243,808 bp. We further refer to these selected regions as regions 1, 2, and 3.

Each of the three regions showed significant correlations to the relative abundance profiles of one of our inferred lineage variants (Fig. 5, line plots). Region 1 highly correlated with the relative abundance profile of variant 1 (95% CI, 0.9731 < *r* < 0.9986; *P* = 6.3E−09), indicating that it was present in that lineage but absent from variants 2 and 3. The genes in region 1 included multiple metabolism-related genes, but its overall functional significance was difficult to assess. Region 2 correlated with the relative abundance profile of variant 3 (95% CI, 0.8447 < *r* < 0.9913; *P* = 8.3E−06). This region contained pyoverdine synthetases and genes from the type VI secretion system. We observed that two SNVs in the region mapped to variant 1, suggesting that it is present in all three lineage variants but amplified at least 2-fold in variant 3. Several dozen paired-end reads spanned from the end of the region back to its start, suggesting the additional copies in variant 3 are either arranged as tandem duplications or form an excised plasmid. Finally, the average coverage of region 3 correlated very well with the combined relative abundance profile of variants 2 and 3 (95% CI, 0.9713 < *r* < 0.9985; *P* = 8.2E−09), indicating that it was absent from variant 1. This region contained multiple genes of the *psl* operon, which plays a role in biofilm generation in P. aeruginosa. Taken together, it becomes clear that the information contained in the relative read coverage over time, when correlated with the relative proportions of SNVs, allows precise and confident mapping of large-scale genomic structure variants to their respective lineage variants.

## DISCUSSION

In this study, we sought insights into the temporal dynamics of the lung microbiome in CF by using noninvasive DNA sequencing of lung sputum. By repeated sampling of the lung microbiome over several months, we were able to distinguish persistent pathogens from other, more transient community members. The taxonomic identification of pathogens from sequencing data were generally in line with the clinical microbiology laboratory reports, although in the case of *Achromobacter* and *Pseudomonas*, the sequence-based identifications proved to be more precise. Moreover, with P. aeruginosa in patient CFR11, at least three distinct lineage variants were observed. Importantly, distinguishing these variants would not have been possible without the longitudinal repeated samplings, showing that tracking patients over time provides valuable added information. To our knowledge, only two other shotgun metagenomics studies with multiple reference points per CF patient have been published (20, 40). Both studies perform strain typing for recognized pathogens but do not explore longitudinal genomic variation on a sublineage level.

Longitudinal data from patient CFR11 allowed us to delineate at least three lineage variants with distinct temporal dynamics for one of the best-covered pathogens, P. aeruginosa. Multiple studies have described genotypically and/or phenotypically distinct P. aeruginosa subpopulations (38, 41–48) and provided insights into their abundance fluctuations over time (43, 48) by sequencing cultured isolates from CF patients’ lungs. Shotgun metagenomics sequencing approaches have indicated that P. aeruginosa is polymorphic in some patients (17, 18), as are some other CF pathogens (17, 18, 49); however, these studies were limited to a single time point for most patients, providing no insight into subpopulation dynamics. Outside CF, in a more controlled *in vitro* setting, a conceptually similar approach to ours has been used to study the molecular evolution of E. coli populations over 60,000 generations, leading to the recognition of coexisting clades (50). Here, we provide a proof of principle that this is also possible in a clinical setting, *in vivo*, without prior knowledge of which pathogen strains to expect in a patient.

The emergence of phenotypically and genotypically distinct subpopulations of P. aeruginosa in CF through lineage diversification has previously been shown to be driven by spatial heterogeneity (44, 51). Lung regions differ in oxygen and carbon dioxide concentrations (52), patterns of ventilation and deposition (53), and disease burden (54). General microbial community composition differs depending on lung region as well (55, 56). Nevertheless, other studies have found no clustering of P. aeruginosa isolates based on region of isolation (57) or have shown identical phenotypes and genotypes in upper and lower airways (58, 59). Sputum sequencing does not provide us with information on the spatial distribution of our lineage variants within the lung, but we have observed strong temporal changes in the relative variant abundances over the course of the study. These could be reflective of shifts in the lung compartments sampled in the sputum or be indicative of general shifts in the complete lung, an interesting question to explore in future research.

Lineage diversification within a patient makes infections in CF an unclear example of strain mixing, as in the case of fecal microbiota transplantation (60). Thus, methods that rely on all subpopulations being represented in a reference database would provide limited insights (61–63). Multiple tools, however, have been developed to reconstruct haplotypes based on genetic variation with or without a reference (30, 60, 64–68). Tools assessing variation in a set of marker genes (60, 65, 67), while allowing subpopulation identification when diagnostic SNVs happen to be present in these markers, preclude insights into subpopulation-specific mutations in other genes that could be of potential interest due to adaptation to the particular lung environment. MetaPalette does use the entire genome (66), but it is unclear whether its “k-mer painting” approach would be able to discern and reconstruct distinct sublineages that differ only by about 1 in 10,000 nucleotides. Of the remaining tools, to our knowledge only EVORhA (64) has been used in a clinical setting (69). This tool explicitly reconstructs haplotypes from reads mapped against a reference, but it does not use the information in longitudinally related samples, instead focusing on abundance differences within each single sample. Moreover, EVORhA has been criticized for artificially inflating the number of haplotypes detected (68–70), including by a study that also used PacBio sequencing for validation (70). Very recently, a promising new method for haplotype reconstruction was published, displaying better performance on synthetic benchmarks and strain mixtures than existing tools (71). This tool (mixtureS) likewise only works on samples individually, but it does employ an expectation maximization algorithm for the final step in strain identification. It has not yet been tested on highly similar lineages in a time course setting, however.

Our approach to longitudinal CF microbiome tracking using short-read metagenomics data still has a number of limitations. Linking SNVs from the whole genome predominantly based on allele frequencies can be obscured by recombination events and the presence of mobile genetic elements. Moreover, SNV linkage requires sufficient data in terms of the number of time points and in terms of sequence read coverage depth. Although we have also performed variant calling and SNV clustering on patients CFR07 and CFR09, the smaller number of time points prevented us from performing lineage deconvolution on P. aeruginosa in a manner similar to that for CFR11. In patient CFR06, *Achromobacter* was covered sufficiently for variant calling at only one time point. Finally, although we had *Achromobacter* data from five time points in patient CFR11, clustering of SNVs showed no apparent sublineages.

We could not perform subpopulation analysis of *A. insuavis* in a manner similar to that with P. aeruginosa, but we could detect an apparent case of clinical species misidentification in both patients CFR06 and CFR11. The observed pathogen lineage likely belongs to *A. insuavis*, not A. xylosoxidans. The misidentification of *Achromobacter* species by conventional clinical methods is not uncommon (26, 72, 73) due to the difficulty of distinguishing species based on 16S rRNA sequence alone (26, 74, 75) and lack of representative spectra in matrix-assisted laser desorption ionization–time-of-flight (MALDI-TOF) databases commonly used by clinical microbiology laboratories (73, 76). Genotyping of several CF patient cohorts using *Achromobacter*-specific marker sequences (26, 72) has revealed *A. insuavis* was the second-most prevalent species after A. xylosoxidans (73, 77–80) or at least accounted for a considerable fraction of *Achromobacter* infections (72). *A. insuavis* is also one of the few *Achromobacter* species capable of chronic infection (77, 79, 80), and our observations in patient CFR11 are in line with previous findings. Overall, our results from P. aeruginosa and *A. insuavis* show that clear and reliable pathogen identification at various taxonomic resolutions is possible without the need for cultivation based on community-wide sequencing data alone.

The limited availability of genetic data for characterization was partly due to an excess of human DNA; up to 93% of generated reads mapped to the human genome, which is not unexpected in studies of lung sputum (17, 18). To enrich for nonhuman material, we performed depletion of methylated DNA. The depletion worked to some extent based on data from paired samples, and we obtained more than 25% nonhuman reads in some samples, which is more than a 2-fold improvement on the numbers from previous studies (17, 18). However, it did not work equally well for each sample. A recent assessment of human DNA depletion methods in human saliva samples showed the limited effectiveness of currently available kits and introduced a new depletion method that decreased the fraction of human reads to 8.53% (81). This method has yet to be applied to sputum. Another recent study proposed a microfluidics-based method to enrich microbial DNA in samples from human airways (82). The implementation of methods enriching for nonhost material in oral and sputum samples looks promising, as this would lead to a decrease in sequencing costs and provide more sequencing material to study the less abundant bacteria.

In general, due to the lack of absolute abundance data, we also cannot be certain whether the observed change in the relative abundance of a specific bacterium could be in response to other bacteria growing and/or dying. In addition, as no explicit dead cell depletion has been performed, some changes in relative abundance could be influenced by the presence of DNA from dead cells, which have been known to accumulate in CF mucus (83). Absolute quantification has already provided novel insights into the gut microbiome (84), and, more recently, the application of quantitative PCR for the absolute quantification of bacteria CF lung microbiome has challenged the existence of a CF lung microbiome in early childhood (85). Combined with WGS, these quantitative approaches present a promising venue to increase interpretability in future studies.

In conclusion, we have demonstrated how metagenomics sequencing of time series data in CF patients can complement routine clinical diagnostics. Combined with recent advances in targeted depletion of human material in samples (81, 82), sequencing costs might sink soon to a point that would allow routine use of workflows such as ours in the clinic; a recent case report estimated a similar procedure would take less than 48 h (40). Noninvasive, whole-genome sequencing of sputum can provide better taxonomic resolution for pathogens than the current methods routinely used in the clinic. Unlike 16S rRNA sequencing, classification can be made on a sublineage level. In addition, by using data from multiple time points, multiple lineage variants of the same species can be tracked within a given patient, including the assignment of variant-specific SNVs and variant-specific large-scale genomic changes. Coupled to a growing database of previously observed strains (ideally including the results of past antibiotics resistance tests as well as clinical outcomes), precise computational lineage identification should enable continuous improvements in monitoring pulmonary infections in CF and assist in making decisions on disease management.

## MATERIALS AND METHODS

### Sputum sample collection.

A cohort of 11 CF patients was monitored over the course of 2 years. All study participants provided informed consent. The study was approved by the Cantonal Ethics Committee, St. Gallen (EKSG 13/112). For the study, participants collected spontaneously produced sputum either at home on the same morning as their doctor consultation or directly at the hospital. All participants have been trained since childhood on how to provide sputum for clinical analysis and were particularly encouraged to brush their teeth and drink water prior to sputum collection. The sputum samples were collected at the Cantonal Hospital St. Gallen, weighed, and aliquoted into sterile tubes. Sputum samples from cohort patients who exhibited extreme clinical phenotypes during the course of the study were selected to undergo shotgun metagenomics sequencing.

### Clinical microbiology pathogen identification.

All samples were subjected to standard clinical microbiology procedures used for CF sputum in an ISO 15089 certified laboratory. Sputum samples were preprocessed with a liquefying agent (Copan SL-solution; RUWAG, Bettlach, Switzerland) before streaking on agar plates. Columbia, chocolate, MacConkey, and CNA agars (Becton, Dickinson, Allschwil, Switzerland) were streaked to support growth of the bacterial spectrum present in the upper airways. For the specific detection of CF-associated pathogens, selective chromogenic plates (bioMérieux, Geneva, Switzerland) were incubated: PAID agar for P. aeruginosa, SAID agar for S. aureus, and BCSA for *Burkholderia* species (*Achromobacter* species usually grow well on this agar as well). All plates were visually inspected after 16 to 24 h of incubation at 36°C with or without 5% (vol/vol) CO_2_ per standard protocol (86), followed by a second inspection after another day of incubation. Colonies suggestive of CF-associated pathogens or showing indicative growth on selective media were subjected to MALDI-TOF analysis on a Bruker MALDI Biotyper (Bruker Daltonics, Bremen, Germany) using the standard direct smear protocol. Per manufacturer recommendations, species identification was considered reliable at a score above 2.000. In cases where no CF-associated pathogen was seen after both inspections, the culture was reported as respiratory tract flora.

### DNA extraction, treatment, and sequencing.

After dilution in Sputolysin (Calbiochem Corp., San Diego, CA, USA), total DNA was extracted using the High Pure PCR template preparation kit (Roche, Basel, Switzerland) per the manufacturer’s instructions. DNA concentration was measured using an ACTgene UV99 spectrophotometer at a wavelength of 260 nm, and samples were stored at −20°C. As the starting material was not limiting and sufficient amounts of DNA were available, no extra amplification step was deemed necessary, and no extraction blanks for PCR/sequencing contamination control were processed.

After DNA isolation, samples were subjected to methylated DNA depletion using the NEBNext microbiome enrichment kit (New England Biolabs Inc., Ipswich, MA, USA) to enrich for microbial DNA. As a control, we included day 0 samples from all patients without performing depletion. Depletion of methylated DNA did not have a consistent effect on the total number of reads obtained (data not shown). Relative microbial DNA content increased in three out of four patients by up to 2.3-fold but did not exceed 27% (data not shown).

Next-generation sequencing libraries were prepared using the TruSeq DNA Nano library preparation kit (Illumina, Inc., CA, USA) per the manufacturer’s instructions. The libraries were sequenced using the Illumina HiSeq 4000 platform (Illumina, Inc., CA) in paired-end mode (2 × 125 bp). Reads were quality checked with FastQC (87).

### Removal of the host genome reads, contig assembly, and annotation.

Reads were aligned to human genome build 38 (88) using BowTie2 (version 2.3.1) (89), reporting at most one alignment per read and writing read pairs that did not align concordantly to a separate file. Reads that did not align concordantly to the human genome were used for downstream analysis and assembly. We assembled reads into contigs using metaSPAdes (version 3.10.1) (90) with the metagenomic sample data flag. The contigs were then searched against the NCBI nucleotide database (as of 24 June 2017) using BLASTn (version 2.6.0) (91). During the search, an E value cutoff of 1E−15 was used, and the five closest matching sequences were retained. For taxonomic annotation, we only considered matching sequences that had a bit score within a 10% range of the maximum scoring match. Contigs were assigned to the most recent common ancestor of the considered matches. Assembly completeness and contamination were assessed using the lineage workflow in CheckM (23). Phages and viruses were largely excluded from this analysis due to their poor representation in databases and lack of a standardized taxonomy.

### Taxonomic profiling and diversity estimation.

Raw reads were trimmed and filtered based on quality using sickle (version 1.33) (92). Trimmed and filtered reads were profiled using mOTUs (version 2.0.1) (profile at molecular operational taxonomic unit [mOTU], genus, and family taxonomic level; output scaled read counts) (22) and MetaPhlAn (version 2.7.1) (profile at all taxonomic levels) (93). For MetaPhlAn input, all trimmed and filtered reads were pooled in the same file. The two methods exhibited several disagreements in species delineation, but the generated taxonomic profiles (compared on a sample-by-sample basis) highly correlated at the genus level (see Data Set S1 at https://string-db.org/suppl/Dataset_S1_Strain-resolved_Microbiome_Dynamics_in_Cystic_Fibrosis.xlsx, mOTUs MetaPhlAn comparison).

The amount of viral and fungal content was estimated with MiCoP (repository cloned August 2020) (21). The run-bwa.py script was used first to map trimmed and filtered reads to the viral and fungal databases provided by the authors. Viral and fungal contents were then profiled using the compute_abundances.py script with default detection thresholds to call organisms as present. Results were output as raw counts.

To determine the aerobe and anaerobe content, detected species were mapped to oxygen tolerance data from BacDive (as of August 2019) (94). Unclassified species from a known genus were labeled as aerobe or anaerobe only when all species of this genus were labeled as aerobes or anaerobes. Otherwise, the label “unknown” was assigned.

Diversity was calculated based on relative abundances obtained from mOTUs using Shannon’s diversity index.

### Strain identification with PanPhlAn.

For A. xylosoxidans, a total of 22 genomes and their annotations were downloaded from the Integrated Microbial Genomes and Microbiomes Database (as of May 2018) (95). These genomes were used to create a pangenome using PanPhlAn (version 1.2.3.6) (28). We used the pooled trimmed and filtered reads as input to the PanPhlAn software to generate gene family presence/absence profiles for both sample and reference genomes, setting the strain similarity percentage threshold to zero to show results from all reference genomes. To call gene family presence, default thresholds were used.

For P. aeruginosa, a total of 2,226 genomes were downloaded from the *Pseudomonas* Genome Database (as of July 2018) (96). Because of the large number of genomes, we could not use the complete set of genomes for PanPhlAn and had to generate a set of representative genomes. Pairwise genomic distances were calculated using the Mash (version 2.0) sketch and dist commands (97). We discarded outlier genomes with an average distance of more than 0.1 and with less than 90% estimated completeness according to BUSCO (version 3.0.2), using the *Gammaproteobacteria* OrthoDB v9 database and the Augustus E. coli gene prediction model (98, 99). Remaining genomes were clustered at a distance threshold of 0.005. From each cluster, we selected the genome with the smallest average distance to all other cluster members, yielding 359 representative genomes. The representative genomes were annotated using Prokka (version 1.12) (100) and used to generate the pangenome using PanPhlAn (version 1.2.3.6) (28) in the same manner as that for A. xylosoxidans.

### Phylogenetic tree generation.

A total of 145 genomes from the *Achromobacter* genus were downloaded from the NCBI Genome database (as of November 2018) (24) but one was discarded due to low estImated completeness. We searched these genomes using BLASTn (version 2.6.0) (91) with an E value cutoff of 1E−15 against the mOTUs database (version 2.0.1) and against the PubMLST database of the *Achromobacter* genus (as of July 2017). For our samples, we searched the contigs assigned to the *Achromobacter* genus against the same databases. We used the coordinates output by the search to extract the corresponding gene sequences from the genomes. If the extracted gene sequence was shorter than the sequences of this gene in the databases, we padded the gene sequence with “X.” If a gene was absent from the genome, we introduced a string of X’s that was the length of this gene.

We then produced two types of composite sequences. Based on the mOTUs database search, sequences were created by concatenating the single-copy genes *COG0012*, *COG0016*, *COG0018*, *COG0172*, *COG0215*, *COG0495*, *COG0525*, *COG0541*, *COG0533*, and *COG0552*. Based on the PubMLST database search, sequences were created by concatenating the housekeeping genes *eno*, *gltB*, *lepA*, *nrdA*, *nuoL*, *nusA*, and *rpoB*. Composite sequences that were more than half X’s were omitted from further analysis. The remaining sequences were aligned using MUSCLE (version 3.8.1551) (101). Based on the alignments, maximum likelihood trees were constructed using RAxML (version 8.2.10) (102) under the GTRCAT model (random seed 1234). Bordetella pertussis (NC_002929.2) was used as an outgroup to root the trees. One hundred bootstraps (random seed 1234) were performed on the trees to estimate branch confidence.

For the third tree, the downloaded *Achromobacter* genomes and/or sample contigs were annotated using Prokka (version 1.12) (100). The obtained gene sequences were used as the input for the ANIcalculator (version 1.0) (103). Genes annotated as rRNA, tRNA, or tmRNA were excluded from the calculation. Based on the calculated pairwise average nucleotide identities, a distance matrix was created and genomes were clustered using the unweighted pair group method with arithmetic mean (UPGMA) function in the R package phangorn (104). The number of monophyletic clades was calculated using the check_monophyly function in the ETE Toolkit (version 3.0) (105).

For the 16S rRNA *Achromobacter* tree, rRNA sequences were predicted using barrnap (version 0.9) for the *Bacteria* kingdom, using the default E value of 1E−06 and rejecting all sequences that were less than 80% of the length threshold (106). In genomes with multiple predicted 16S rRNA sequences, we selected the sequence that had the highest average alignment score across all predicted singleton 16S rRNA sequences. Furthermore, we discarded the sequences from *Achromobacter* sp. strain KAs 3-5 and *Achromobacter* sp. strain BFMG1, as a quick search revealed these sequences were from a different family. A total of 127 sequences (including B. pertussis) were used to generate the alignment and tree using the same procedure as that for the *Achromobacter* mOTUs tree. The Robinson-Foulds metric was calculated using the compare function in the ETE toolkit (version 3.0) (105). Only genomes present in all trees were considered for the comparison.

The P. aeruginosa tree in Fig. S5D was generated using the same procedure as that for the *Achromobacter* mOTUs tree. No outgroup was used during tree generation, but midpoint rooting was used during tree visualization. All trees were visualized using iTOL (107).

### Calling single-nucleotide variations.

Filtered and trimmed sample reads were mapped to the P. aeruginosa PAER4_119 (CP013113.1) and A. xylosoxidans FDAARGOS_147 (CP014060.1; data not used further) genomes using the ngless framework provided by the developers of metaSNV (version 0.8.1) (108–111). The framework filtered out reads that did not map uniquely, mapped at an identity of less than 97%, or had less than a 45-bp match with the reference. SNVs were called using metaSNV (version 1.0.3) (29), under default thresholds.

### Comparison of SNV temporal profiles from the clusters in Fig. 4.

To investigate the relationship between the temporal profiles of the seven SNV clusters, an SNV from each considered cluster was drawn at random. Allele frequencies from all 10 time points were added or subtracted between drawn SNVs in accordance with the claims made, and the mean absolute error relative to the expected value (0 or 1) was calculated. A total of 10,000 draws were performed. To generate a random distribution, one of the drawn SNVs had the time points shuffled prior to performing arithmetic operations. The real and random mean absolute error distributions were compared to each other using a two-sided Kolmogorov-Smirnov test.

Temporal variation distributions were generated by using the standard deviation of allele frequencies from 10 time points for each SNV within the considered t-SNE cluster. The seven clusters were then pairwise compared using a two-sided Mann-Whitney U test. A Bonferroni correction was then applied to the obtained *P* values.

### Haplotype detection with DESMAN.

Prior to running DESMAN (version 2.1.1) (30), we filtered out SNVs that clustered together on the *P. aeruginosa* PAER4_119 chromosome (more than 8 per 1,000 bp), because these could have biased the relative abundance calculations. The Variant_Filter.py script was used to further select SNVs, resulting in 1,287 SNVs used by DESMAN to determine the relative abundance for three haplotypes. Ten runs, each consisting of 100 iterations, were performed using different random seeds (1 to 10).

### Long-read sequencing and analysis.

DNA isolated from day 94 and day 346 samples of patient CFR11 was additionally subjected to long-read sequencing. The sequencing libraries were prepared using the SMRTbell Express template preparation kit 2.0 (Pacific Biosciences of California, Inc.).

Prior to sequencing, size selection was performed on the DNA. Fifteen micrograms of genomic DNA (gDNA) was mechanically sheared to an average size distribution of 10 to 20 kb using a Megaruptor 3.0 device (Diagenode) and a Femto pulse gDNA analysis assay (Agilent). Ten micrograms of sheared gDNA was DNA damage repaired and end repaired using polishing enzymes. A ligation and a nuclease treatment reaction were performed to create the SMRT bell template per the manufacturer’s instructions. A Blue Pippin device (Sage Science) was used to size select the SMRT bell template and enrich the big fragments that were longer than 8 kb. A ready-to-sequence SMRT bell-polymerase complex was created using the Sequel II binding kit 2.0 and Internal Control 1.0 (Pacific Biosciences of California, Inc.) per the manufacturer’s instructions.

The Pacific Biosciences Sequel II instrument was programmed to sequence the library on 1 Sequel II SMRT Cell 8M (Pacific Biosciences of California, Inc.), taking one 30-h movie per cell, using the Sequel II sequencing kit 2.0 (Pacific Biosciences of California, Inc.). After the run, read quality was assessed using the “run QC” module in the PacBio SMRT Link software.

Reads with an average quality above 20 were mapped to the P. aeruginosa PAER4_119 (CP013113.1) genome using minimap2 (version 2.17-r941) (112) with “PacBio vs reference mapping” preset parameters. We selected reads that mapped to the reference with a sequence identity of at least 97% and spanned at least two variant-specific SNVs. Diagnostic reads with haplotypes not matching any of the three variants were labeled “incompatible.” For the expected read count estimation, haplotypes for each diagnostic read were drawn randomly in proportion to the allele frequencies output by metaSNV for the corresponding sample. The procedure was repeated 10,000 times to generate a background distribution.

### Analysis of P. aeruginosa genome coverage.

Read mapping was performed as described in “Calling single-nucleotide variations,” above. Average read coverage was calculated for windows of 1,000 bp. To account for higher coverage near origins of replication (39), we fit a quadratic polynomial function to every sample’s coverage profile. Each window’s coverage was then normalized to the value output by the function at the chromosome position in the middle of the window. The circos diagram in Fig. 5 was generated using the R package circlize (113).

### Phage region detection.

Locations of phage sequences on the P. aeruginosa PAER4_119 (CP013113.1) genome were determined through the use of multiple tools: PHASTER (114), Phage Web (115), Phigaro (116), and PhiSpy (117, 118). For both PHASTER and Phage Web, the web interface was used to search for the GenBank accession number (as of August 2020). We kept the default Phage Web settings for phage region identification: at least 80% sequence identity during BLAST, at least six coding sequences in a prophage region, and 80% of the elements in identified prophage regions to be used for integrity analysis. Phigaro (version 2.2.5) was run locally in basic mode. PhiSpy (version 4.1.17) was run locally three times using different training sets, genericAll, *Pseudomonas*, and 208964.452 (based on the P. aeruginosa PAO1 genome), with otherwise default parameters. Between all tools and runs, 17 potential regions were detected, although only five regions were detected by more than one tool. We adjusted phage region boundaries based on the overlap between multiple tools and by looking at alterations in read coverage. An additional sixth phage region was selected because it was detected in all PhiSpy runs and displayed similar coverage abnormalities.

### Data availability.

All samples, after removal of reads that mapped to the human genome, have been deposited in the European Nucleotide Archive (PRJEB32062). Data Set S1, which includes study information gathered for each patient (clinical information, prescribed medication, lung microbiome taxonomic profiles, and assembly reports) and a list of P. aeruginosa genes containing lineage variant-specific SNVs, can be downloaded at https://string-db.org/suppl/Dataset_S1_Strain-resolved_Microbiome_Dynamics_in_Cystic_Fibrosis.xlsx.
