# Supplementary material for: Strain-Resolved Dynamics of the Lung Microbiome in Patients with Cystic Fibrosis
Source: mBio. 2021 Mar 9;12(2):e02863-20. doi: 10.1128/mBio.02863-20 (PMC8092271; doi:10.1128/mBio.02863-20)
Supplement: FIG S1 [file mBio.02863-20-sf001.pdf]

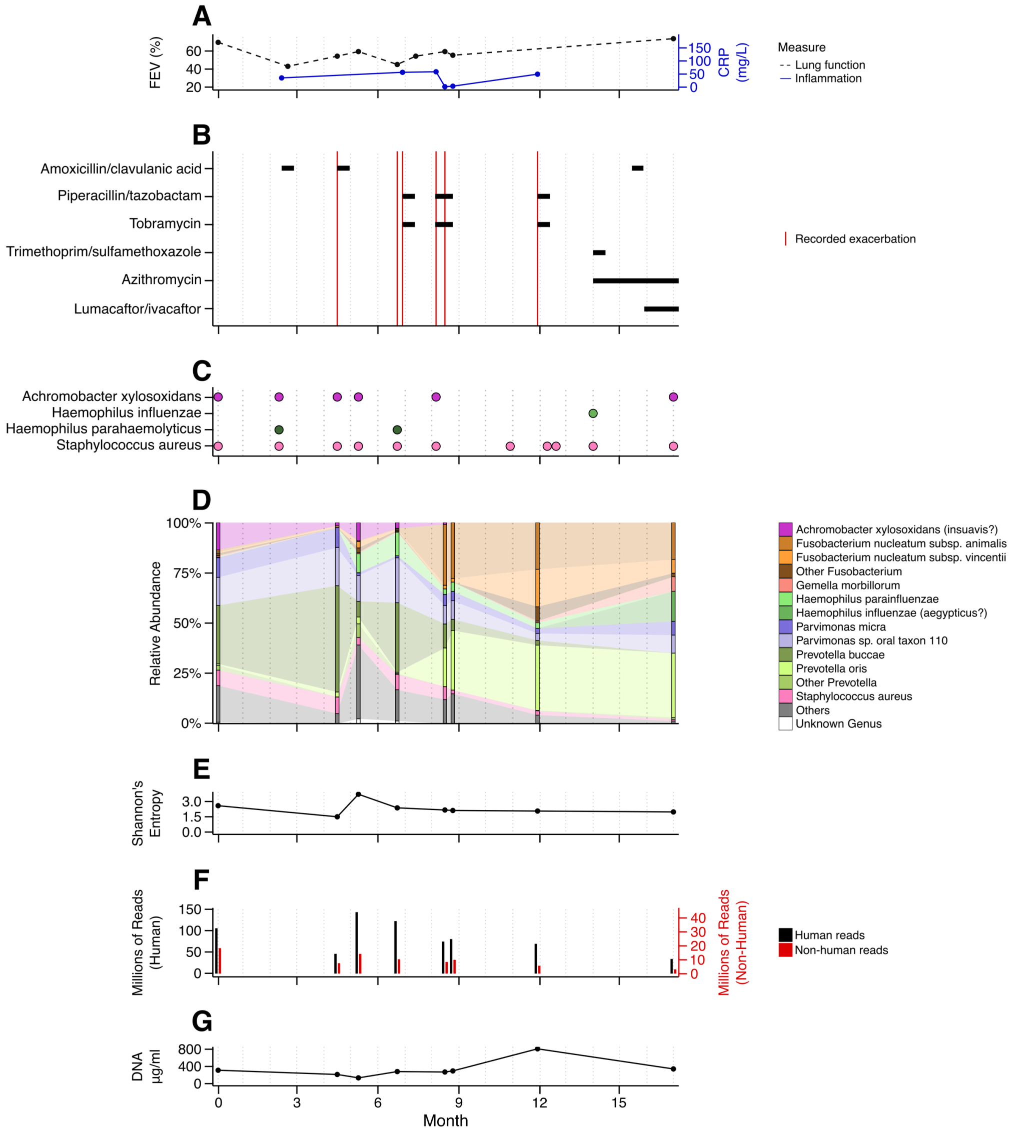

Figure S1. Study report of patient CFR06 displaying the dynamics of multiple parameters over time. (A) percentage forced expiratory volume (FEV) (black) and concentration of C-reactive protein (CRP) (blue), with actual measurements shown as dots. **(B)** Medication assigned to the patient during the course of the study and recorded exacerbation events (in red). **(C)** Bacteria identified in the clinical microbiology laboratory. **(D)** Relative abundance profiles generated by mOTUs, with actual measurements shown as bars. Selected species and their corresponding genera with more than 5% relative abundance at at least one time-point are shown color-coded. Less abundant species are grouped into “Others” (grey). Taxa that could not be identified by mOTUs on the genus level are grouped into “Unknown Genus” (white). **(E)** Shannon’s entropy calculated based on the relative abundance profiles generated by mOTUs, with actual measurements shown as dots. **(F)** Number of reads per sample. Human reads are indicated in black and plotted on the left axis. Reads that did not concordantly map to the human genome are indicated in red and plotted on the right axis. **(G)** Concentration of total DNA isolated from patient sputum, with actual measurements shown as dots.
