# Supplementary material for: Strain-Resolved Dynamics of the Lung Microbiome in Patients with Cystic Fibrosis
Source: mBio. 2021 Mar 9;12(2):e02863-20. doi: 10.1128/mBio.02863-20 (PMC8092271; doi:10.1128/mBio.02863-20)
Supplement: FIG S4 [file mBio.02863-20-sf004.pdf]

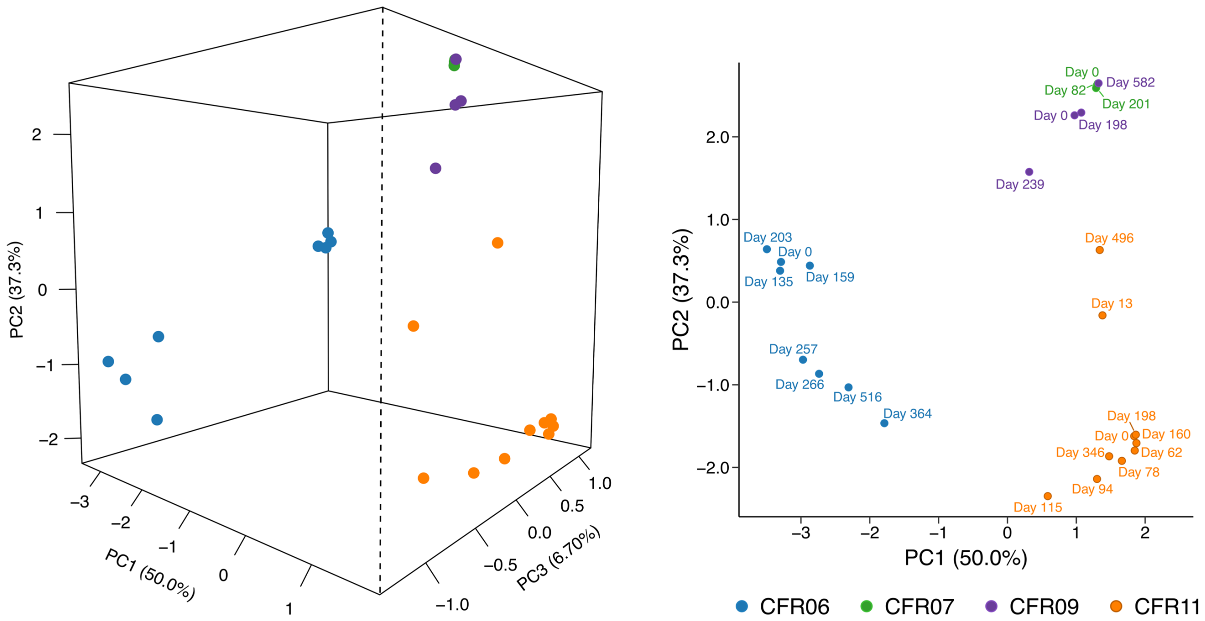

Figure S4. Principal component analysis of Manhattan distances between patient samples. The plot on the left shows the first three components (94% of the variation). The plot on the right shows the first two components (87% of the variation) with time points at which the samples were collected. Colors: CFR06 (blue), CFR07 (green), CFR09 (purple), CFR11 (orange).
