# Supplementary material for: Strain-Resolved Dynamics of the Lung Microbiome in Patients with Cystic Fibrosis
Source: mBio. 2021 Mar 9;12(2):e02863-20. doi: 10.1128/mBio.02863-20 (PMC8092271; doi:10.1128/mBio.02863-20)
Supplement: FIG S5 [file mBio.02863-20-sf005.pdf]

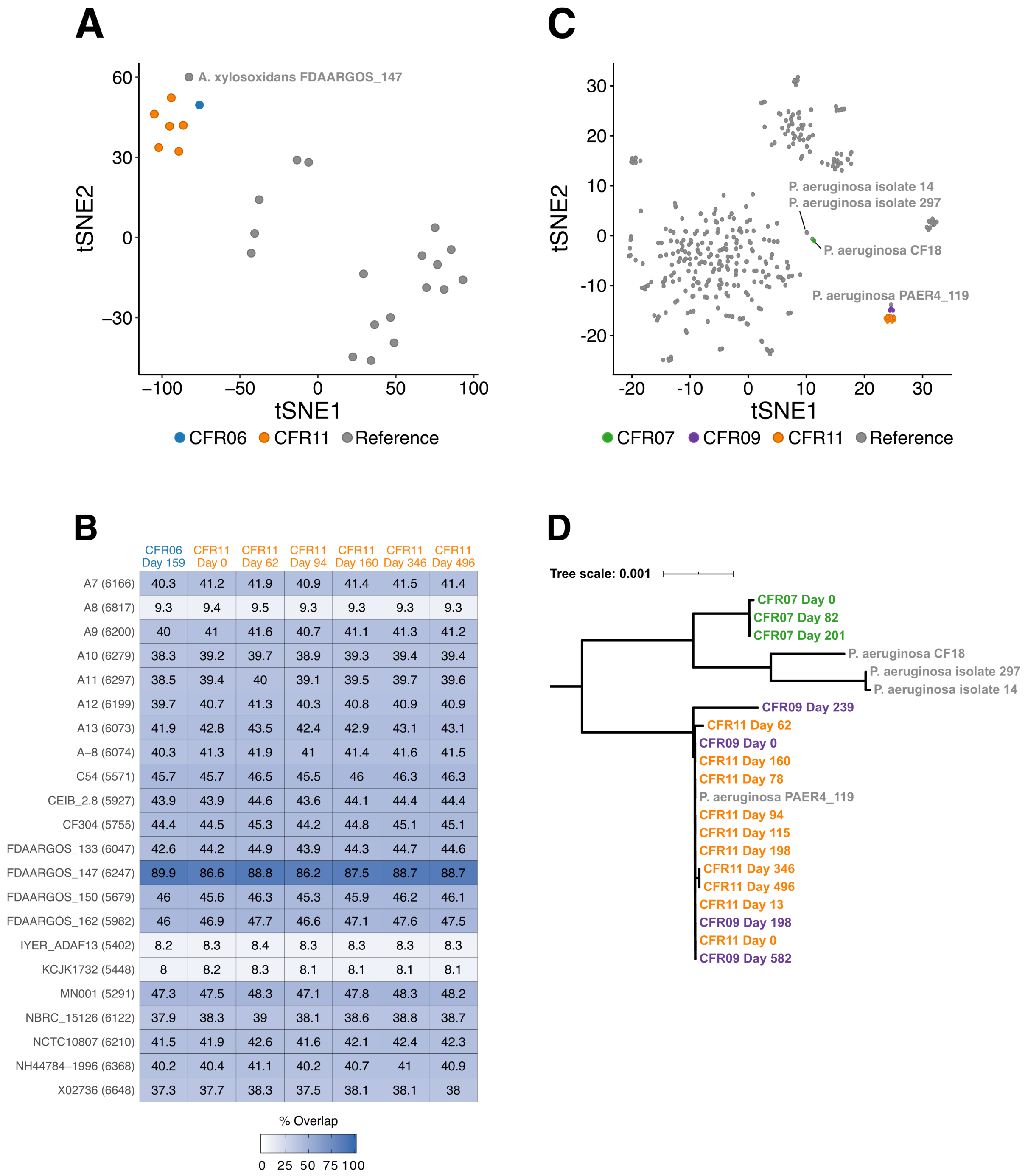

Figure S5. Strain identification for clinically relevant pathogens *A. xylosoxidans* and *P. aeruginosa*. (**A**) t-SNE plot based on *A. xylosoxidans* gene-family presence/absence profiles generated by PanPhlAn. **(B)** Heat-map showing the percentage of gene family overlap calculated based on the gene-family presence/absence profiles from **(A).** **(C)** t-SNE plot based on P. aeruginosa gene-family presence/absence profiles generated by PanPhlAn. **(D)** Midpoint-rooted phylogenetic tree of selected *P. aeruginosa* strains based on ten gene marker sequences from mOTUs. Colors: CFR06 (blue), CFR07 (green), CFR09 (purple), CFR11 (orange), reference genomes (grey).
