# Supplementary material for: Strain-Resolved Dynamics of the Lung Microbiome in Patients with Cystic Fibrosis
Source: mBio. 2021 Mar 9;12(2):e02863-20. doi: 10.1128/mBio.02863-20 (PMC8092271; doi:10.1128/mBio.02863-20)
Supplement: FIG S6 [file mBio.02863-20-sf006.pdf]

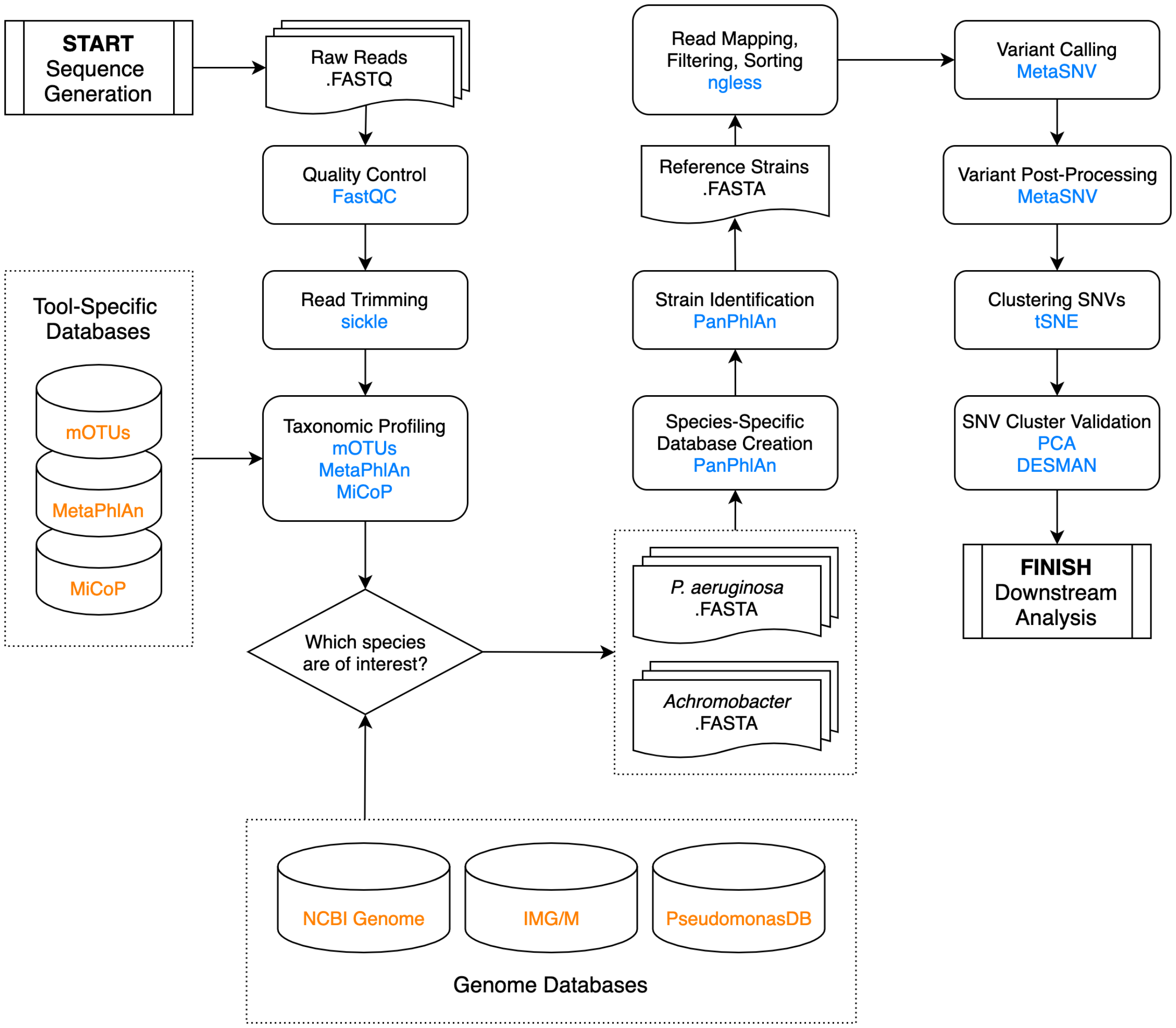

Figure S6. A flow-chart depiction of steps performed to identify lineage variants in the study. The names of databases used in the analysis are depicted in orange. The names of tools used in the analysis are depicted in blue.
