# Supplementary material for: Strain-Resolved Dynamics of the Lung Microbiome in Patients with Cystic Fibrosis
Source: mBio. 2021 Mar 9;12(2):e02863-20. doi: 10.1128/mBio.02863-20 (PMC8092271; doi:10.1128/mBio.02863-20)
Supplement: FIG S7 [file mBio.02863-20-sf007.pdf]

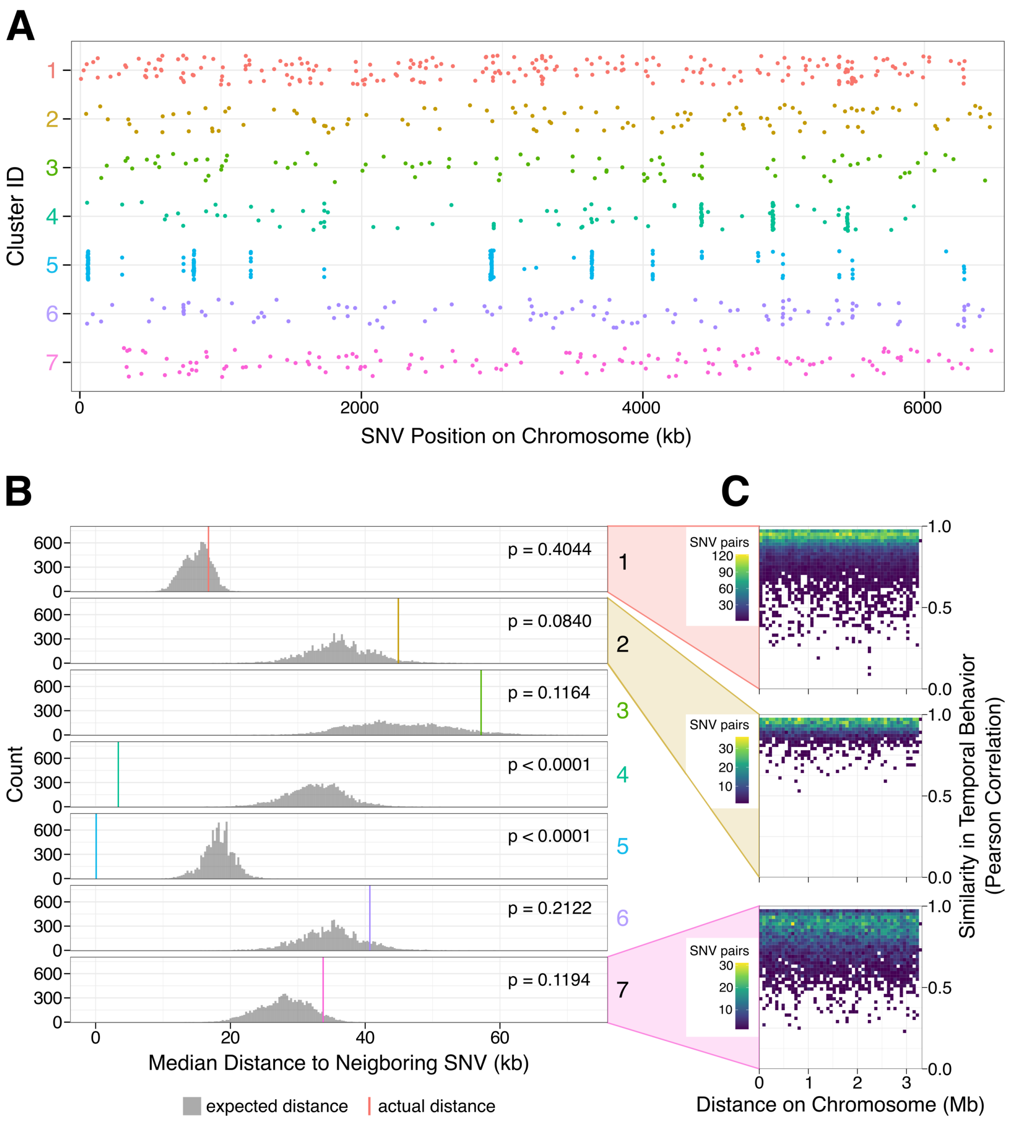

Figure S7. Assessment of positional bias in the distribution of SNVs from the Fig. 4 SNV clusters from patient CFR11 in the *P. aeruginosa* genome. (A) Plot depicting the distribution of SNVs along the *P. aeruginosa* PAER4_119 (CP013113.1) chromosome. Each dot represents an SNV assigned to one of the seven clusters and is colored according to its cluster in Fig. 4. **(B)** Quantification of positional bias in the distribution of SNVs on the chromosome. Vertical lines indicate the actual median distance between neighboring SNVs. Background distributions of median distances between neighboring SNVs (in grey) are generated by randomly selecting SNV groups of the same size as the considered cluster from the seven-cluster SNV pool. The procedure is repeated 10,000 times for each cluster. P-values reflect the probability of the actual distance lying within the background distribution. **(C)** Temporal profile similarity of SNV pairs versus the distance between these SNVs (for selected clusters #1, #2, and #7). Results are represented as a 2D histogram in which yellow indicates a high number of SNV pairs and blue indicates a low number of SNV pairs. No significant correlations between temporal profile similarity and chromosomal distance are found.
