# Supplementary material for: Strain-Resolved Dynamics of the Lung Microbiome in Patients with Cystic Fibrosis
Source: mBio. 2021 Mar 9;12(2):e02863-20. doi: 10.1128/mBio.02863-20 (PMC8092271; doi:10.1128/mBio.02863-20)
Supplement: FIG S8 [file mBio.02863-20-sf008.pdf]

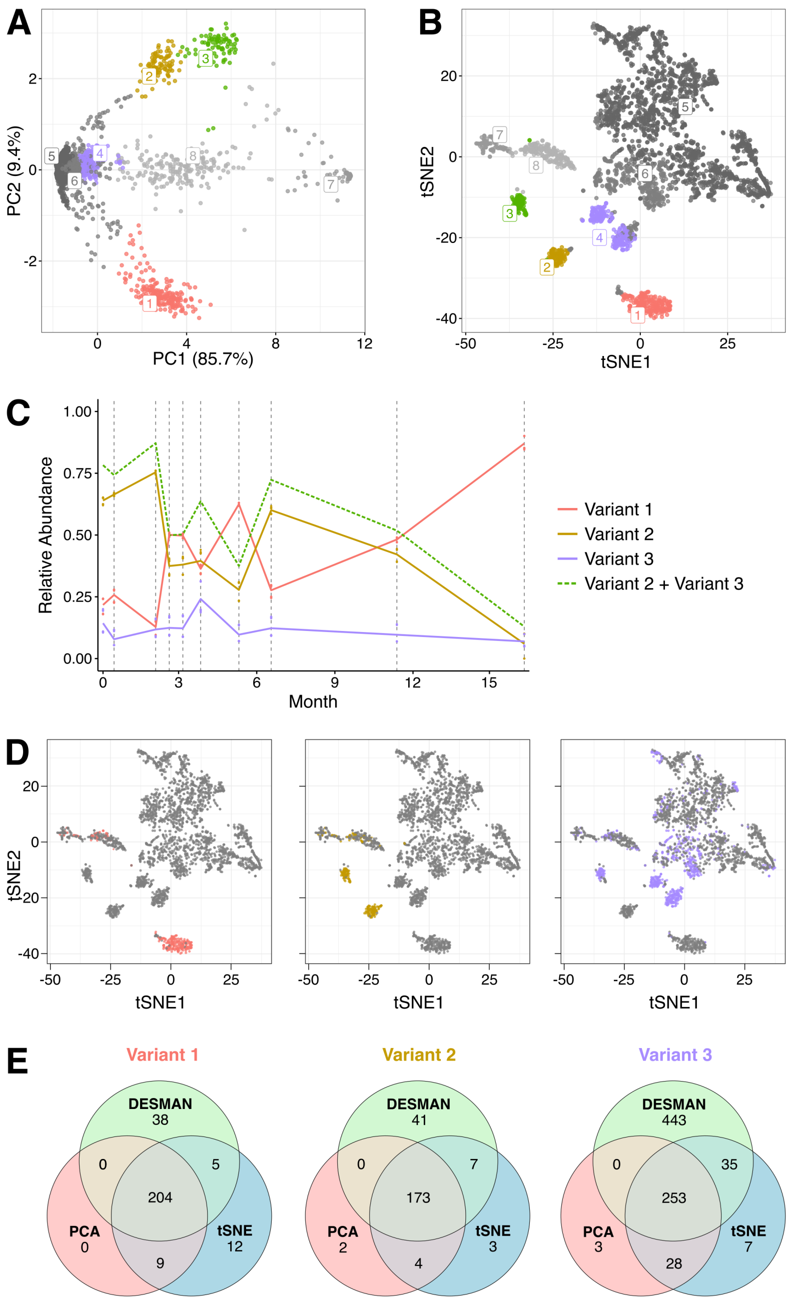

Figure S8. Validation of detected *P. aeruginosa* SNV clusters in patient CFR11. (A) Plot of the first two principal components (95% of the variation) generated based on the complete allele frequency table. SNVs are clustered into eight clusters based on the first three components (97% of variation), and dots are colored according to cluster assignment. **(B)** Projections of the clusters shown in **(A)** on the t-SNE plot from Fig. 4. **(C)** Temporal relative abundance profiles of the three lineage variants detected by DESMAN. Dots depict individual runs (n = 10) and the line shows the average value. **(D)** Projection of SNVs assigned to each of the three lineage variants by DESMAN on the t-SNE plot form Fig. 4. Colors indicate lineage variant assignment: red (variant 1), yellow (variant 2), purple (variant 3). **(E)** Venn diagrams depicting the overlap of SNVs assigned to each of the three variants by t-SNE clustering, principal component clustering, and DESMAN.
