# Supplementary material for: Strain-Resolved Dynamics of the Lung Microbiome in Patients with Cystic Fibrosis
Source: mBio. 2021 Mar 9;12(2):e02863-20. doi: 10.1128/mBio.02863-20 (PMC8092271; doi:10.1128/mBio.02863-20)
Supplement: FIG S9 [file mBio.02863-20-sf009.pdf]

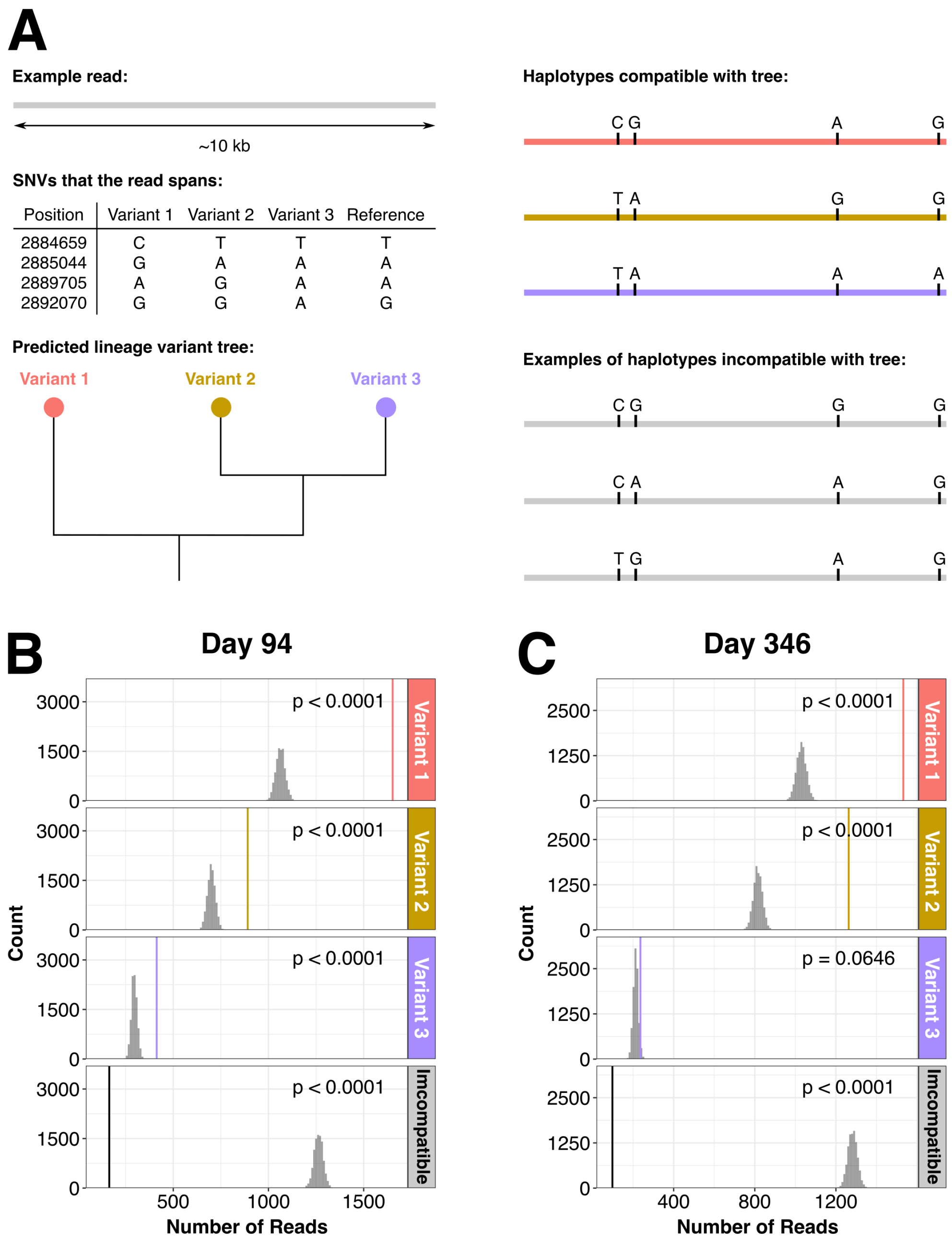

Figure S9. Validation of detected *P. aeruginosa* lineage variants in patient CFR11 via long-read sequencing. (A) An example of a read spanning four SNVs that are diagnostic for specific lineage variants. A haplotype is assigned based on the combination of base pairs at the indicated positions in the read. This haplotype is either compatible with one of the proposed lineage variants or labeled “incompatible”. The number of observed long reads corresponding to each defined haplotype can be seen in **(B)** for the Day 94 sample and **(C)** for the Day 346 sample. Vertical lines indicate the observed number of reads. Background distributions of read numbers (in grey) are created by generating random haplotypes based on metaSNV allele frequencies from the corresponding day. The procedure is repeated 10,000 times for all reads spanning at least two diagnostic SNVs. P-values reflect the probability of the observed number of reads lying within the background distribution.
