# Supplementary material for: Strain-Resolved Dynamics of the Lung Microbiome in Patients with Cystic Fibrosis
Source: mBio. 2021 Mar 9;12(2):e02863-20. doi: 10.1128/mBio.02863-20 (PMC8092271; doi:10.1128/mBio.02863-20)
Supplement: FIG S10 [file mBio.02863-20-sf010.pdf]

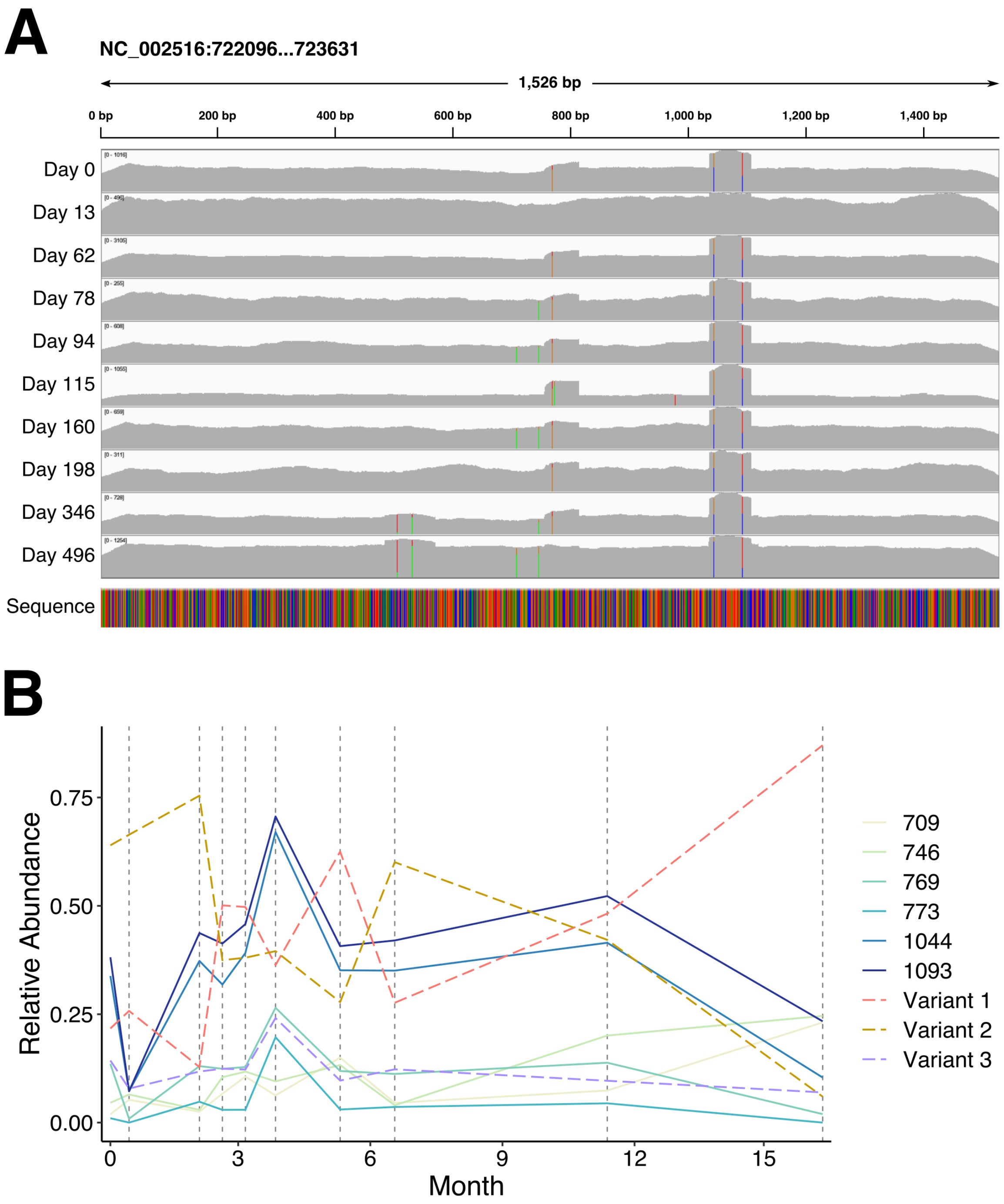

Figure S10. SNV detection in the 16S rRNA sequence of *P. aeruginosa*. (A) A snapshot from Integrative Genome Viewer showing 16S rRNA coverage profiles from all time points of patient CFR11. Reference sequence shown on the bottom in color: adenine (green), cytosine (blue), guanine (orange), thymine (red). Deviations from the reference sequence shown as colorful stripes on the coverage profiles. **(B)** Temporal dynamics of the fraction of reads containing detected SNVs. SNVs are depicted in different colors, the number corresponding to location in the 16S sequence. The relative abundances of the three lineage variants detected by DESMAN are shown as dotted lines. SNVs at positions 769, 773, 1004, and 1093 are located in regions with higher coverage and closely resemble the gain in coverage.
